# Single shot detection of alterations across multiple ionic currents from assimilation of cell membrane dynamics

**DOI:** 10.1038/s41598-024-56576-3

**Published:** 2024-03-12

**Authors:** Paul G. Morris, Joseph D. Taylor, Julian F. R. Paton, Alain Nogaret

**Affiliations:** 1 Department of Physics, University of Bath, Claverton Down, Bath, UK; 2 School of Physiology, Pharmacology and Neuroscience, University of Bristol, Bristol, UK; 3 Manaaki Manawa − the Centre for Heart Research, Department of Physiology, Faculty of Medical and Health Sciences, University of Auckland, Grafton, Auckland, New Zealand

**Keywords:** Ion transport, Nonlinear phenomena

## Abstract

The dysfunction of ion channels is a causative factor in a variety of neurological diseases, thereby defining the implicated channels as key drug targets. The detection of functional changes in multiple specific ionic currents currently presents a challenge, particularly when the neurological causes are either a priori unknown, or are unexpected. Traditional patch clamp electrophysiology is a powerful tool in this regard but is low throughput. Here, we introduce a single-shot method for detecting alterations amongst a range of ion channel types from subtle changes in membrane voltage in response to a short chaotically driven current clamp protocol. We used data assimilation to estimate the parameters of individual ion channels and from these we reconstructed ionic currents which exhibit significantly lower error than the parameter estimates. Such reconstructed currents thereby become sensitive predictors of functional alterations in biological ion channels. The technique correctly predicted which ionic current was altered, and by approximately how much, following pharmacological blockade of BK, SK, A-type K^+^ and HCN channels in hippocampal CA1 neurons. We anticipate this assay technique could aid in the detection of functional changes in specific ionic currents during drug screening, as well as in research targeting ion channel dysfunction.

## Introduction

The complement of ion channels in a cell membrane underpins key aspects of neuronal function such as the shape of action potentials^1^, adaptive versus non-adaptive firing response^2^, integration of synaptic inputs^3,4^ and some forms of short or long-term memory^5^. Dysfunction of a single channel type can substantially alter cellular behaviour: for example, channelopathies, in which certain ion channels are either absent or exhibit abnormal conductances, are known to be the causative factor in forms of epilepsy^6,7^, pain disorders^8^, cystic fibrosis^9^, and cardiac arrhythmias^10^. Channelopathies also form part of a more complex pathophysiology in Parkinson’s^11,12^ and Alzheimer’s^13^ diseases, Rett syndrome^14^, and autism^15^. Such outcomes drive the development of efficient methods for detecting ion channel dysfunction. In relation to disease, channelopathies may arise from changes in ion channel density, expression, gene mutations, and loss of function, such as in autoimmune disease^16,17^. Mutational channelopathies are identified in research by high throughput sequencing, or patch-sequencing which aims to correlate morphological and electrical alterations to underlying gene mutations^18^. These sequencing approaches face challenges in identifying disease-linked mutations, particularly in autoimmune diseases where channel function may be altered by factors beyond the mutations themselves. Patch-clamp electrophysiology is the primary technique used for profiling ion channels in vitro, both in research studies investigating ion channel dysfunction in disease models, and in screening candidate drugs targeting ion channels. However, in both scenarios this approach is low throughput and labor-intensive^19^, particularly if neurological causes are a priori unknown or involve multiple ionic currents. A single shot method is therefore highly desirable which can reconstruct ionic currents from multiple channel types from the effects they induce in the electrical response of a neuron. This would have the benefit of removing guess work by inferring changes across all ion channels simultaneously and by mapping the range of genes encoding altered subunits to the exclusion of all others whose effects are unknown.

In this work, we demonstrate a powerful method based on statistical data assimilation (DA) that extracts information on multiple ionic currents simultaneously from chaotically driven current-clamp recordings. The method synchronizes a Hodgkin–Huxley-like model^20^ to the membrane voltage oscillations of a hippocampal neuron to estimate ion channel parameters^21–24^ such as maximal conductances^25^, voltage thresholds, slopes of activation curves, and recovery time constants that constitute the fingerprints of individual ion channels^26–28^. The predictive power of our approach is based on the observation that ionic currents reconstructed from estimated parameters carry an uncertainty three times lower than the parameters themselves. We subsequently use the ionic charge transferred per action potential as a reliable metric to predict ion channel alterations induced by channel antagonists. We find that changes in predicted ionic charge match the selectivity and potency of well-characterized inhibitory compounds applied to block BK, SK, A-type K^+^, and HCN channels. This approach is to our knowledge unique in inferring actual changes across a range of ion channel types from subtle changes in membrane voltage dynamics. The method can be applied to primary tissue, including animal models of disease, rather than being limited to cell cultures. The statistical readouts indicate any changes across the range of ionic currents simultaneously in the cell of interest in response to a drug or treatment. This method may prove beneficial in early drug screening, and in research studies aiming to detect functional changes amongst a wide range of ionic currents.

## Results

### Statistical data assimilation of pharmacologically altered neurons

**Figure 1:**
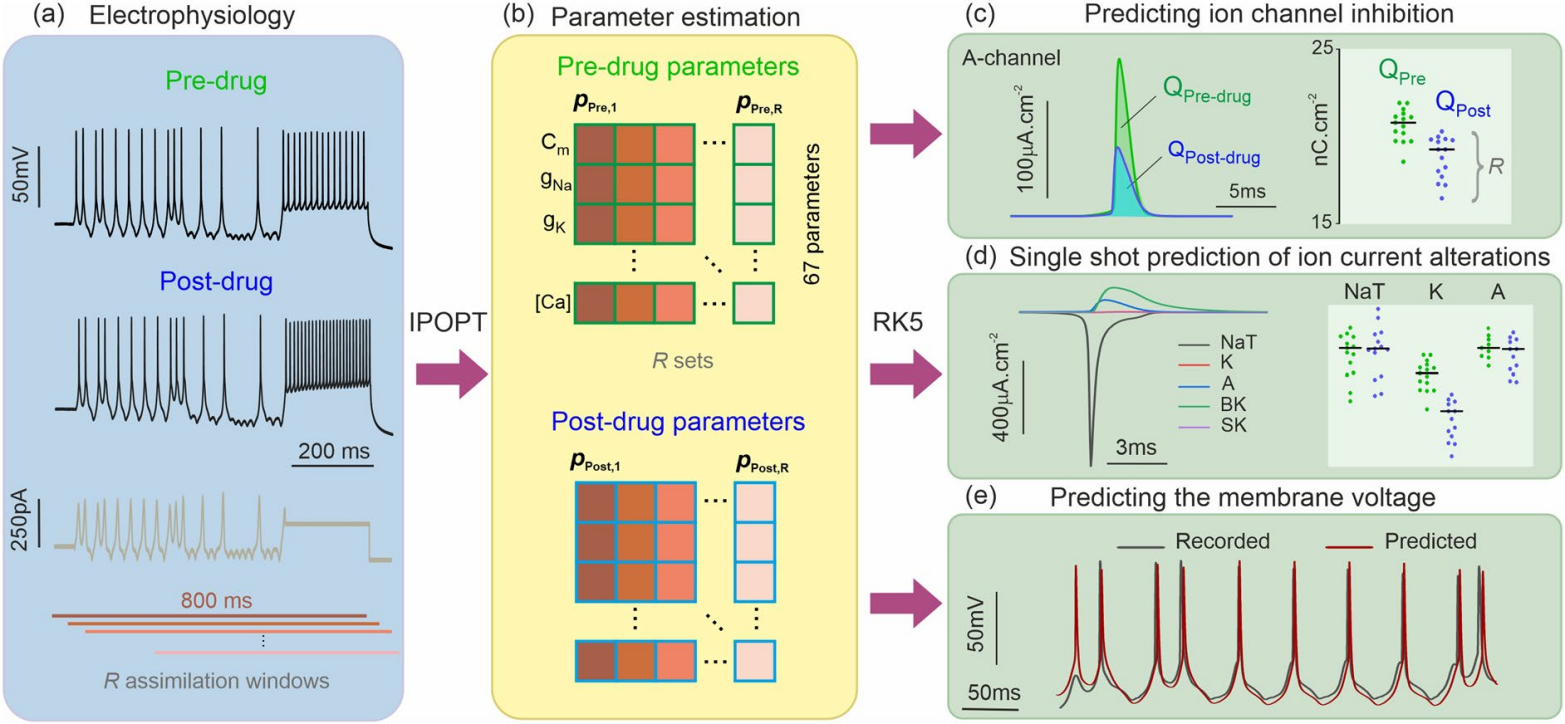
Estimation of ion current alterations from current clamp recordings. (**a**) Membrane voltage of a (hippocampal) neuron recorded before and after a pharmacological inhibitor is applied to partially block a specific ion channel (black traces). The same current protocol (brown trace) is applied to elicit pre-drug and post-drug oscillations. (**b**) Data assimilation (IPOPT) was used to synchronize a nine-ion channel Hodgkin–Huxley model to the data over a 800 ms long time window and obtain one set of pre-drug parameters {p^*^_Pre_} and one set of post-drug parameters {p^*^_Post_}. Each set has *K* = 67 parameters. This approach was repeated over *R* assimilation windows offset by 80 ms to generate a statistical sample of parameter sets {p^*^_Pre_}_1,…,*R*_ and {p^*^_Post_}_1,…,*R*_ where *R* = 15–19 depending on the antagonist applied. (**c**–**e**) The Hodgkin–Huxley model configured with each set of estimated parameters was used to predict the ionic current waveforms and membrane voltage oscillations through forward integration of the current protocol with an adaptive step-size fifth order Runge–Kutta method (RK5). (**c**, **d**) The degree of channel block was predicted by calculating the amount of ionic charge transferred per action potential, $Q_{Pre - drug}$ and $Q_{Post - drug}$, for all nine ion channels of the model. Predictions were validated by comparing the median and mean reductions in charge transfer to the known selectivity and potency of the antagonist. (**e**) Predictions were also validated by comparing the predicted membrane voltage to the measured one.

The statistical DA workflow is schematically depicted in Fig. 1. The membrane voltage of a hippocampal CA1 neuron is recorded whilst being driven by a chaotic sequence of current waveforms designed to elicit hyperpolarizing and depolarizing responses across many time scales and amplitudes (Fig. 1a). For each experiment the current-clamp protocol was applied twice: first in the natural state and a second time after applying an antagonist to block a specific ion channel (Fig. S1). We then synchronized the neuron model to electrophysiological recordings using interior point optimization^29^, a constrained nonlinear optimization framework. The neuron model was a single compartment Hodgkin–Huxley-type system incorporating the 8 ion channels most prevalent in the CA1 soma (Table S1). Each modelled ion channel represents an amalgam of the possible subtypes of that channel: for example, the ‘SK’ channel represents the gating and response dynamics of both SK1 and SK2 subunits. Interior point optimization inferred the 67 parameters that best synchronize the model to electrophysiological data over an 800 ms long assimilation window (Table S2). One set of 67 parameters was obtained from pre-drug data (*p*_*pre*_) and another from post-drug data (***p***_*post*_). Preliminary assimilations of model-generated data successfully recovered the 67 parameters of the original model to within 0.2% and with a 100% convergence rate^21,30^. Convergence was achieved irrespective of starting conditions and the positioning of the assimilation windows in a 2000 ms long epoch. This indicates that the observability^31^ and identifiability criteria^32^ which are necessary to reconstruct the model’s state variables and parameters from measurements, are fulfilled. In contrast, the problem of assimilating biological neuron data is complicated by our lack of knowledge of the exact model. Model error introduces correlations between some parameter estimates. As a result, parameter search tends to converge towards multiple solutions depending on the choice of starting conditions. In order to mitigate the uncertainty on parameters, we generated a statistical sample of parameters ***p***_*pre*,1_… ***p***_*pre*,*R*_ and ***p***_*post*,1_… ***p***_*post*,*R*_ (Fig. 1b) by assimilating *R* windows offset by 80 ms from each other (Figs. 1a). We then completed 2*R* conductance models by inserting the pre-drug and post-drug parameters in the model equations. The ionic current waveforms (Fig. 1c,d) and membrane voltage oscillations (Fig. 1e) were predicted by forward-integrating the stimulating protocol (Fig. 1a) with pre-drug and post-drug completed models. We then numerically integrated the current waveform of each ion channel, to obtain the ionic charge transferred per action potential, pre-drug and post-drug. We repeated this process for the *R* assimilation windows to generate a statistical distribution of the ionic charge transferred (Fig. 1c). All *R* current waveforms were calculated at the site of one action potential chosen for being in the short time interval overlapped by all assimilation windows. The statistical distributions of ionic charges were plotted (Fig. 1c) and analyzed (Mann–Whitney) to estimate the median and mean predicted inhibition for each channel. To ensure that our predictions are not affected by the firing frequency of neurons, which we found to be particularly sensitive to potassium channel inhibition, we calculated the charge transfer at the site of a single action potential instead of over the entire assimilation window. Ionic current waveforms were reconstructed and analyzed in parallel allowing all current alterations to be predicted in one shot (Fig. 1d). Forward integration of the model also generated the predicted membrane voltage time series (Fig. 1e). The agreement between the experimentally observed and the predicted voltage provides an intermediate validation point of our method.

### Accuracy of current and parameter predictions

**Figure 2:**
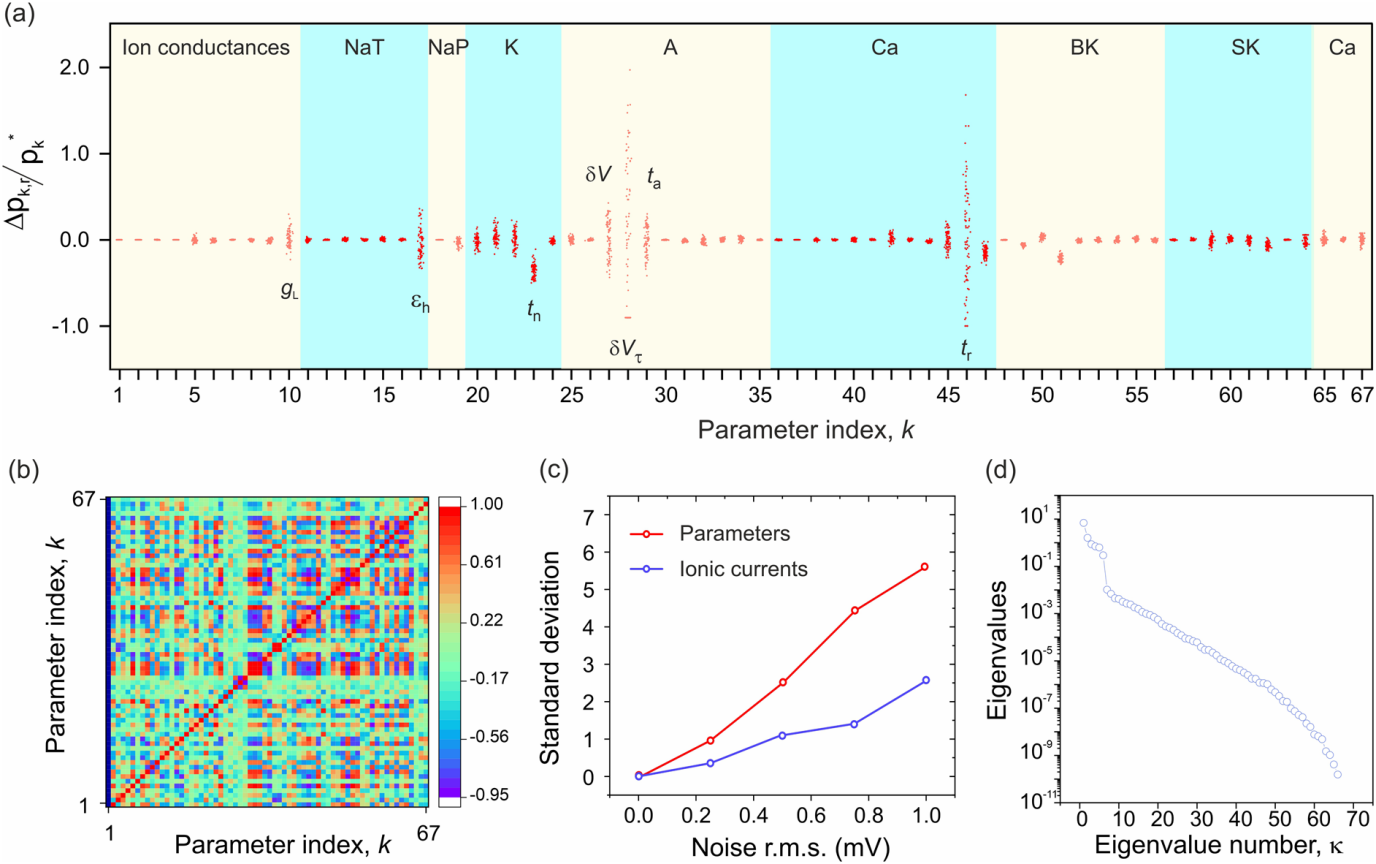
Comparing the uncertainty on estimated ionic currents and parameter. (**a**) Deviations of parameter estimates from their true values ($p_{k}^{*}$), $k = 1 \ldots 67$, when Gaussian noise is added to the membrane voltage. Parameter deviations ${\Delta }p_{k,r} = \left( {p_{k,r} - p_{k}^{*} } \right)$ were computed from a statistical sample of ℜ = 100 assimilations of the same 800 ms window with 100 different realizations of added noise (0.25 mV *r.m.s*.) (red dots). The greater the dispersion, the greater the parameter sensitivity to data (and model) error. (**b**) Covariance matrix of parameter deviations: ${\upsigma }_{{kk^{\prime}}}^{{}} = \frac{1}{\mathcal{R} - 1}\sum\nolimits_{r = 1} {\left( {\frac{{{\Delta }p_{k,r} }}{{p_{k}^{*} }}} \right)\left( {\frac{{{\Delta }p_{{k^{\prime},r}} }}{{p_{{k^{\prime}}}^{*} }}} \right)}$. Correlations occur within blocks of parameters pertaining to the *same ionic current*. In contrast, correlations between the parameters of different ionic currents are weaker. (**c**) Comparison of the standard deviations of predicted ionic currents and of their underlying parameters. The parameter standard deviation (red dot) is an average of the relative standard deviations of the 67 parameters each calculated over the statistical sample of 100 noise realizations. The current standard deviation (black dot) was calculated by integrating the 100 sodium current waveforms over the assimilation window and computing the relative standard deviation of the integral charge. The *uncertainty on ionic currents is three times smaller than on parameters*. (**d**) Spectrum of eigenvalues of the covariance matrix. The 6 outliers determine the 6 directions of parameter correlations in parameter space.

The main challenge to inferring biologically relevant information from actual neurons as opposed to model data is to minimize the error introduced in the parameter field by model error and, to a lesser extent, measurement error^32–34^. In order to quantify the impact of model or data error, we calculated 100 sets of parameters by assimilating model data corrupted by 100 different realizations of white noise (Fig. 2). The parameters that deviate significantly from their true values (Fig. 2a) are few and mainly associated with gate recovery times ($t$, $\epsilon$) (Table S2). In order to clarify the nature of parameter correlations, we calculated the 67 × 67 covariance matrix of this dataset (Fig. 2b). We find that the covariance matrix exhibits a block structure whereby the correlations between parameters pertaining to the same ionic current are greater than those pertaining to different ionic currents. These findings suggest that the greater parameter correlations might compensate each other in the calculation of ionic currents. This underpins our key hypothesis that ionic currents might be calculated with a higher degree of confidence than their underlying parameters. A calculation of standard deviations of ionic currents and parameters over a range of noise levels (Fig. 2c) validates this hypothesis by predicting a three times lower uncertainty on ionic currents. This finding allows us to focus on ionic current as a metric of ion channel alterations, and to validate the magnitude of alterations against the effect of antagonists of known selectivity and potency. Figure 2d plots the eigenvalues of the covariance matrix which measure the lengths of semi-axes of the data misfit ellipsoid. There are six outliers at the left which point to six principal directions along which parameters are very loosely constrained with $\frac{{{\Delta }p}}{p} \approx 100\%$. Along the 61 other principal directions $\frac{{{\Delta }p}}{p}$ varies between 7 and 0.001% confirming that most parameter estimates are well constrained as observed in Fig. 2a. We now use these findings to predict the selectivity and potency of four ion channel antagonists applied to rodent hippocampal neurons.

### Predicting the alterations of ion channels induced by four antagonists in hippocampal neurons

#### BK channel blockade

**Figure 3:**
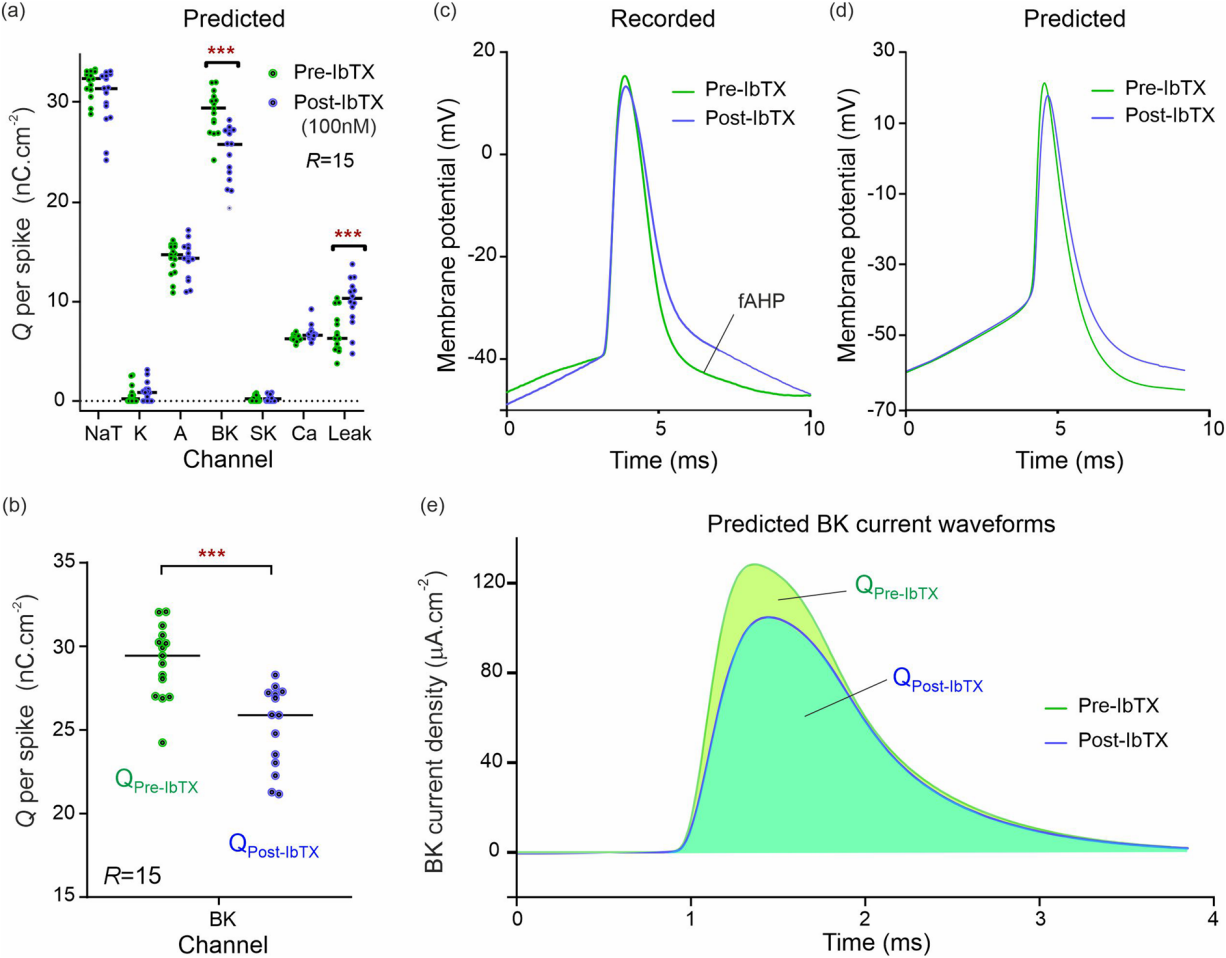
Single-shot prediction of ionic current block by Iberiotoxin (IbTX). (**a**) Predicted ionic charge transferred per action potential, per ion channel, across the complement of ion channels of a CA1 neuron. The green dots are the charge predictions computed from *R* = 15 assimilation windows of pre-drug neuron recordings. The blue dots are the charge predictions computed similarly from the same neuron after 100 nM IbTX was applied. Horizontal bars show median charge values. Asterisks (***) indicate multiplicity adjusted q values from multiple Mann–Whitney U tests using a False Discovery Rate approach of 1%. (**b**) Predicted change in BK charge transfer showing the effect of IbTX as the nominal BK antagonist. (**c**) Effect of IbTX measured in one action potential. Inhibition of the BK channels reduces afterhyperpolarization (fAHP). (**d**) Effect of IbTX predicted for the same action potential. Each voltage trace is the average of 15 waveforms computed from 15 assimilations windows. (**e**) Predicted BK current waveforms and their alteration by IbTX. Each waveform is the average of 15 BK current waveforms reconstructed from 15 assimilation windows.

The analysis of neurons subjected to BK channel blocker iberiotoxin (IbTX; 100 nM; Fig. 3; *R* = 15 pre-drug and post-drug) predicted a *12.1% reduction* in median and 14.8% reduction in mean BK-mediated charge per action potential. This was one statistical discovery across all channels in the drug-applied data (Fig. 3a; U = 25; q < 0.01; mean ranks 21.2 [pre-IbTX], 9.8 [post-IbTX]). Charge transfer decreased from 29.4 nC cm^−2^ to 25.9 nC.cm^-2^. A compensatory increase in leak current was also identified, likely due to decreased K^+^ permeability caused by IbTX (U = 37.5; q < 0.01; mean rank 10.5 [pre IbTX], 20.5 [post-IbTX]). Leak charge transfer increased from 6.3 to 10.3 nC cm^−2^, with a mean increase of 46%. There were no statistical discoveries for any other channels. This demonstrates that models constructed by DA correctly predict the selectivity of IbTX. Figure 3b predicts the reduction in charge transfer through the BK channel targeted by IbTX. Identically driven action potentials measured pre-IbTX and post-IbTX (Fig. 3c) are compared to the action potentials predicted from our pre-IbTX and post-IbTX models (Fig. 3d). The model correctly predicts the reduction in afterhyperpolarization (fAHP) observed post-IbTX. BK current waveforms were also predicted by forward-integration of the pre-IbTX and post-IbTX conductance models (Fig. 3e). The area under both waveforms yielded the drop in BK-mediated charge transfer plotted in Fig. 3b.

#### SK channel blockade

**Figure 4:**
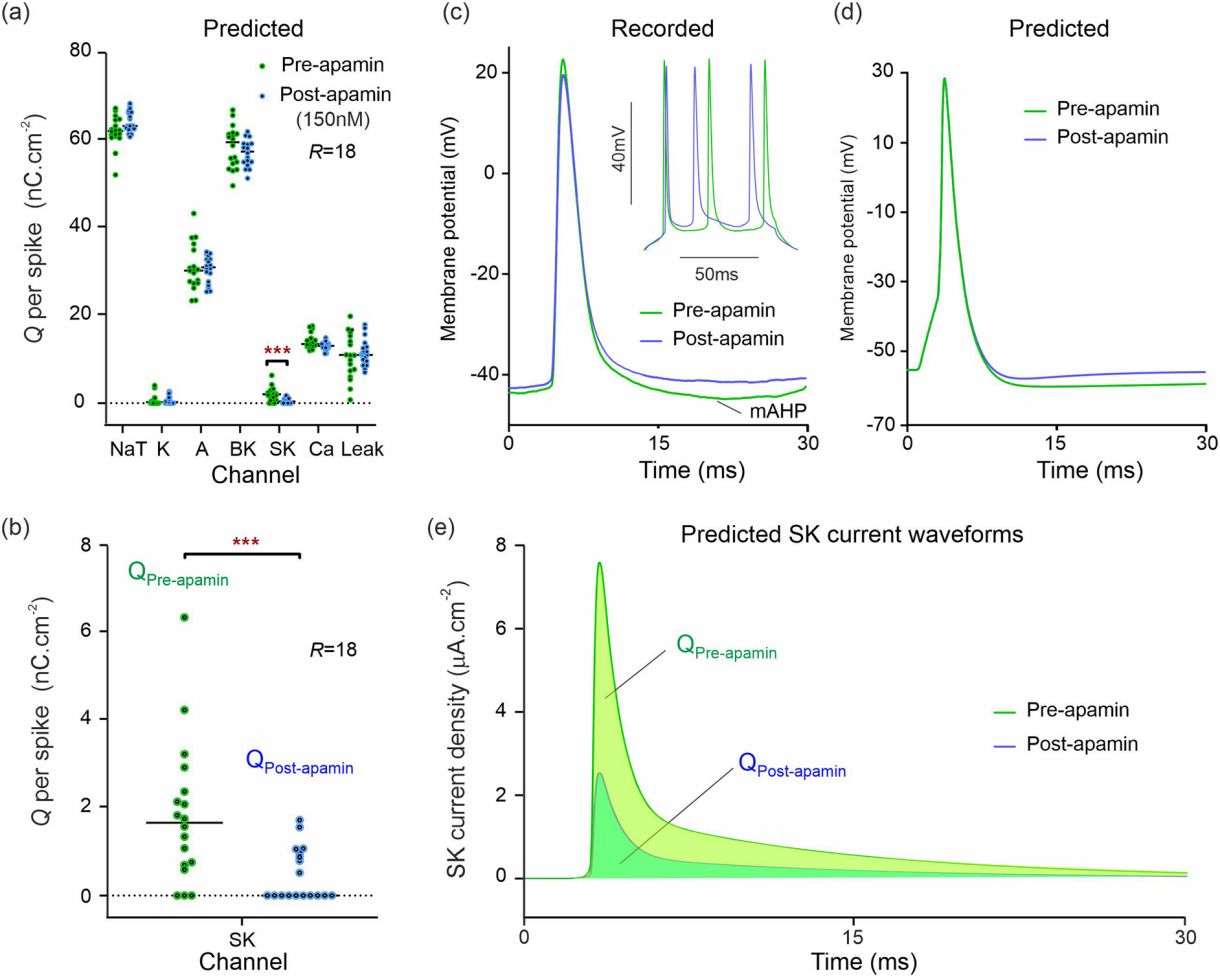
Single shot prediction of ionic current block by apamin. (**a**) Predicted ionic charge transferred per action potential, per ion channel, of a CA1 neuron. The green dots are the ionic charges predicted from *R* = 18 assimilation windows of pre-drug recordings. The blue dots are the predictions computed similarly after 150 nM apamin was applied to the neuron. Horizontal bars are the median charge values. Asterisks (***) represent multiplicity adjusted q values from multiple Mann–Whitney U tests. (**b**) Predicted change in SK charge transfer showing the effect of apamin as the nominal SK antagonist. (**c**) Effect of apamin on one action potential. *Inset*: same for multiple action potentials. (**d**) Effect of apamin predicted for the same action potential. (**e**) Predicted SK current waveforms and their alteration by apamin.

Following application of the SK-specific channel blocker apamin (150 nM; Fig. 4; *R* = 18 pre-drug and post-drug), our model predicted lower SK-mediated charge transfer (Fig. 4a; U = 65; q < 0.01; mean rank 23.9 [pre-apamin], 13.1 [post-apamin]). Median charge transfer dropped from 1.66 nC cm^−2^ to 0, with a mean reduction of 74.0%. This was the sole statistical discovery across all channels in the spike-normalized data. Our model thus correctly predicts that apamin is an antagonist of the SK channel. Figure 4b shows the predicted potency of apamin by plotting the reduction in SK-mediated charge transfer from the pre-apamin state to the post-apamin state. The identically driven action potentials measured pre-apamin and post-apamin (Fig. 4c) are compared to the action potentials predicted by our conductance models (Fig. 4d). The models correctly predict the reduction in medium afterhyperpolarization (mAHP) observed post-apamin in the tail end of the action potential. Forward-integration of the models also predicted the SK current waveforms at the site of an action potential pre- and post-apamin (Fig. 4e). These waveforms were integrated in time to obtain the predicted amounts of SK-mediated charge transfer which were then plotted in Fig. 4b.

#### Kv channel blockade

**Figure 5:**
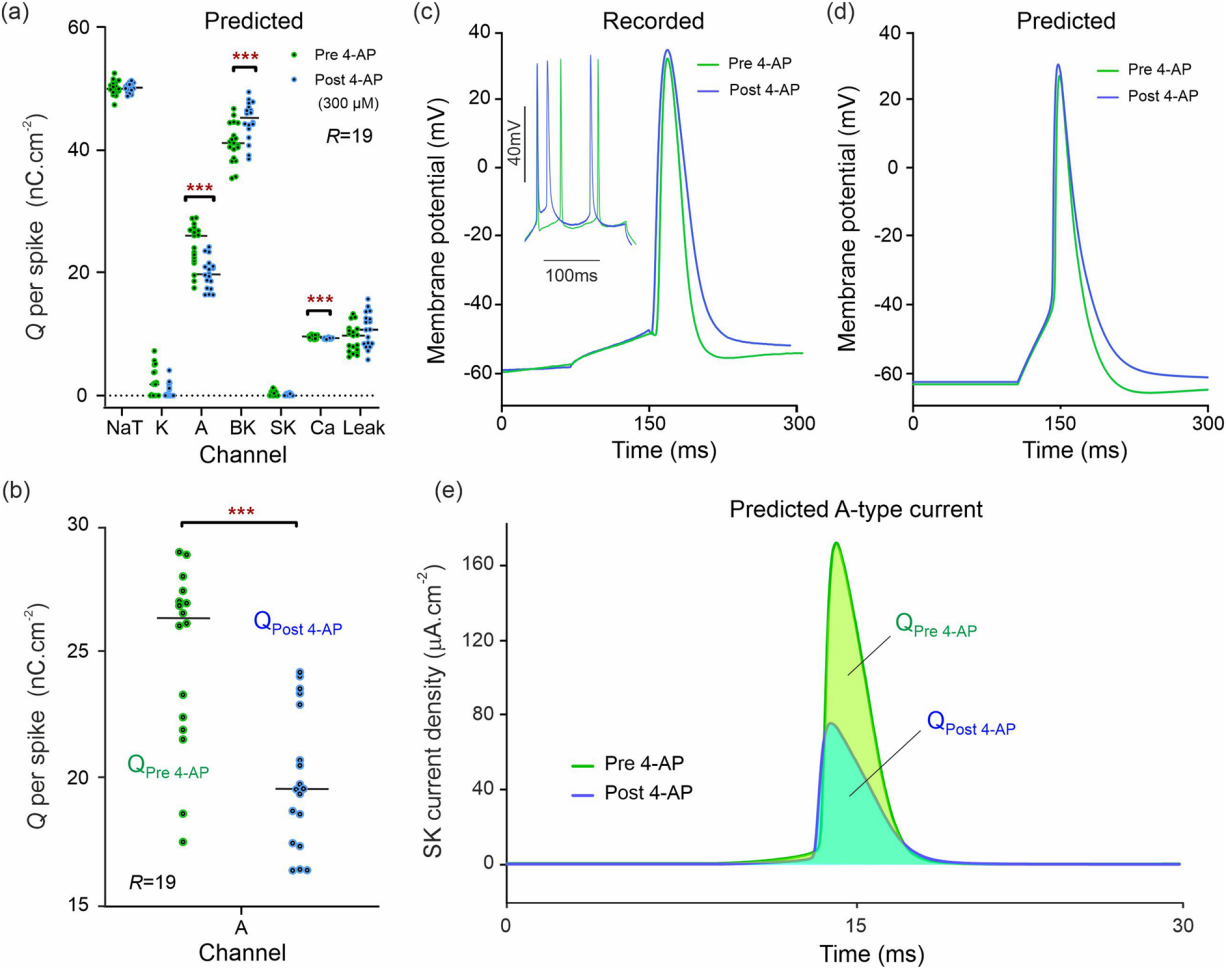
Single shot prediction of ionic current block by 4-Aminopyridine (4-AP). (**a**) Predicted ionic charge transferred per action potential, per ion channel of a CA1 neuron. The green dots show the charge predicted from *R* = 19 assimilation windows of pre-drug recordings. The blue dots show the same after 300 µM 4-AP after was applied to the neuron. (**b**) Predicted change in A-type charge transfer showing the effect of 4-APP as the nominal A-type antagonist. (**c**) Effect of 4-AP on one action potential. *Inset*: 4-AP increases the speed of adaptation of the neuron to stimulation following removal of the A-current-mediated delay. (**d**) Effect of 4-AP predicted for the same action potential. (**e**) Predicted A-type current waveforms and their alteration by 4-AP.

Following application of 4-Aminopyridine (4-AP) to block the voltage-gated potassium channels (300 µM; Fig. 5; *R* = 19 pre-drug; *R* = 18 post-drug), our completed models predicted a reduction in charge transfer mediated by A-type K^+^ channels (Fig. 5a; U = 52; q < 0.001; mean rank 25.3 [pre 4-AP], 12.4 [post 4-AP]). Median charge transfer dropped from 26.1 to 19.7 nC.cm^−2^ with a 19.0% mean reduction. In addition, the model predicts a 10.0% increase in median charge transfer (8.8% mean) through the BK-channel (U = 73; q < 0.01; median charge 41.2 nC cm^−2^ [pre 4-AP], 45.3 nC cm^−2^ [post 4-AP]); and a reduction in Ca^2+^-mediated charge transfer (U = 79; q < 0.01; mean rank 23.8 [pre 4-AP], 13.9 [post 4-AP]). Ca^2+^-mediated charge dropped from 9.65 to 9.23 nC cm^−2^ with a mean reduction of 3.0% mean. Figure 5b predicts the reduction in charge transfer through the A-type K^+^ channels targeted by 4-AP. Action potentials measured pre 4-AP and post 4-AP (Fig. 5c) match the action potentials predicted by our pre 4-AP and post 4-AP models (Fig. 5d). The model correctly predicts the widening of action potentials induced by 4-AP which follows from a slower AHP repolarization. Figure 5e plots the *predicted* A-type K^+^ current waveforms elicited within the same action potential. The predicted current amplitude drops sharply in response to 4-AP. The K^+^ charge amounts transferred per action potential are obtained by integrating the pre 4-AP and post 4-AP current waveforms and plotted in Fig. 5b.

#### HCN channel blockade

**Figure 6:**
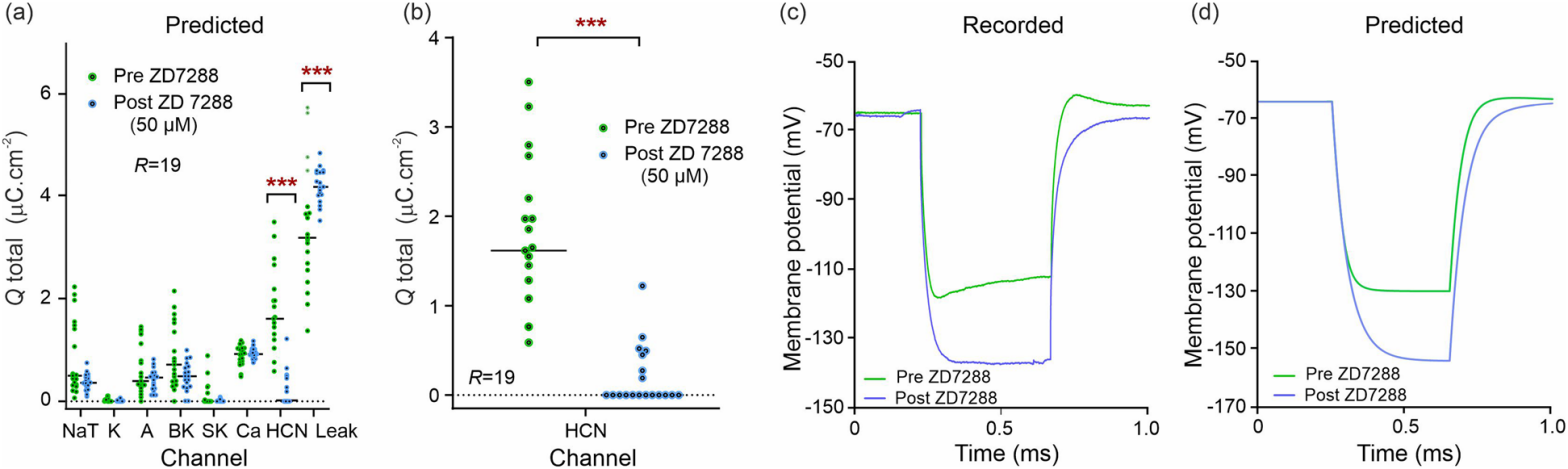
Single shot prediction of the ionic current block by ZD7288. (**a**) Predicted ionic charge transferred per ion channel over an entire 800 ms long assimilation window for a CA1 neuron. The green dots are the charge predicted from *R* = 19 assimilations of pre-drug recordings. The blue dots are the charge predictions computed similarly after 50 µM ZD7288 was applied. (**b**) Predicted change in HCN-type charge transfer showing the effect of ZD7288 as the nominal HCN antagonist. (**c**) Effect of ZD7288 observed during a hyperpolarizing current step activating the HCN channel. (**d**) Effect of the ZD7288 antagonist predicted in response to the same hyperpolarizing step.

We finally applied the ZD7288 antagonist to block the HCN channels (50 μM, Fig. 6; *R* = 19 pre-drug and post-drug). Our completed models predict a reduction in HCN-mediated charge transferred across the full length of the assimilation window (Fig. 6a; U = 81; q < 0.01; mean rank 24.7 [pre-ZD7288], 10.5 [post-ZD7288]). Median charge transfer was reduced from 1.618 µC cm^−2^ [pre-ZD7288] to 0.0 with a mean reduction of 85.5%. In addition, our model predicts an increase in leak current (U = 77; q < 0.01; mean rank 14.1 [pre-ZD7288], 25.0 [post-ZD7288]), with median charge transfer increasing from 3.20 to 4.19 µC cm^−2^ a mean increase of 25.1%. These numbers represent the HCN charge amounts transferred across one 800 ms long assimilation window rather than per action potential as above. This is because the HCN current contributes to subthreshold oscillations unlike the SK, BK and A-type currents which contributes to action potentials. Figure 6b predicts the total blockage of the HCN channel targeted by ZD7288. The membrane voltage response to a hyperpolarizing current step applied before and after ZD7288 (Fig. 6c) is compared to the responses predicted by the pre-ZD7288 and post-ZD7288 models to the same current step (Fig. 6d). The model correctly predicts the faster adaptation and the reduced amplitude of the membrane voltage change post-ZD7288.

In order to validate the results of data assimilation, we now compare the predicted changes in ionic charge transfer to the selectivity and potency of each ion channel antagonist determined by IC50 analysis. The results are summarized in Table 1. The predicted reductions in charge transfer are in good agreement with degree of inhibition expected in SK, BK, A-type and HCN. We further discuss below the inhibition of sub-types of the SK, BK, A, and HCN channels. Besides correctly identifying the selectivity of known antagonists, DA is sensitive enough to pick up correlations between ion channels driven by the modulation of reversal potentials^35^ (Fig. 6a) or compensation mechanisms^36^ (Fig. 5a). We also determined the degree of confidence in our predictions by computing the coefficient of variation (Table 1). The results consistently show a ± 11% uncertainty on charge estimates.

**Table 1:** Validation of the predicted current alterations against the known potency of antagonists.

Drug	Dose (Mol)	Ion channel (sub-unit)		Expected block (Reference)		Median & Mean inhibition	CoV
IBTX	0.1	BK	[α]	86%	^44^	12.1% & 14.8%	21.3%
		BK	[α + β1]	12%	^44^		
		BK	[α + β3]	77%	^44^		
		BK	[α + β4]	Insensitive	^44^		
Apamin	0.15	SK	[mAHP]	~ 100%	^50^	100% & 74.0%	0.1%
		SK2	(+ + +)	~ 100%	^52^		
		SK3	( +)	~ 80%	^52^		
		SK1	(+ +)	Insensitive	^51^		
4-AP	300	Kv1.4	( +)	~ 25%	^57^	24.3% & 19.0%	21.1%
		Kv4.2	(+ +)	~ 13%	^58^		
ZD7288	50	HCN		70%	^68^	100% & 85.5%	24.1%
				85%	^72^		
		HCN1		~ 63%	^65^		
		HCN2		~ 80%	^67^		

The median and mean degrees of inhibition predicted using data assimilation are compared to the known potency of the antagonist at the same concentration as in our experiments. (+)/(++)/(+++) indicates the relative degree of expression (low, medium, high) of individual ion channel sub-types^48–50^. The Kv1.4 and Kv4.2 channels are both A-type.

## Discussion

This proof-of-concept study demonstrates that the DA approach we present can concurrently infer functional alterations across a range of ionic currents. Single hippocampal CA1 neurons in acute brain slices were characterized by driving them with a chaotic current clamp protocol designed to extract the maximum of information for parameter identifiability, and with fast synaptic neurotransmission blocked. The ionic currents were reconstructed with a high degree of confidence from the model parameters estimated by DA. This technique is potentially applicable to assaying multiple ionic currents during functional drug screening, or research studies targeting neurological disease. We now discuss the two factors limiting its predictive accuracy. The first is parameter estimation in the presence of model error. The second is the variation in subunits making up each ion channel, when we are limited to modelling channels using an aggregate contribution of all subunits.

Figures 3a, 4a, 5a and 6a demonstrate the ability of the method to disentangle the contributions of 8 different ionic channels from the membrane voltage time series and to assign drug induced changes to the correct ion channel being blocked. This identification relies on the uniqueness of the mathematical equations of each ionic current in the model (Table S1), and notably the correct ion channel type can still be identified even when we know that model equations only approximate biological reality. There are, however, reasons to believe that model error is merely residual because the completed models make excellent predictions of the membrane voltage pre-drug and post-drug (Figs. 1e, 3d, 4d, 5d, 6d) and because DA assigns stable, sensible values to a majority of parameters. In order to evaluate the effect of model error on current estimates, we deliberately introduced an erroneous gate exponent in the sodium current (NaT), changing the gate exponent $m^{3} h$–$m^{2} h$. We find that the current waveforms estimated with the wrong model deviate only by a few percent from their true shape. We also find any drop in sodium current (NaT) induced by model error is compensated by a drop in potassium (A) current. This indicates that a slightly wrong model still retains its ability to discriminate ion channel types as observed in Figs. 3a, 4a, 5a, 6a. This robustness to model error is important as it allows for the construction of larger, more inclusive models that do not rely on prior assumptions about which ion channel to include, making them applicable to a wide range of biological neurons. As the model size grows, simulations on model data ought to verify the stimulation protocol still satisfies identifiability criteria^31,32^ as a prerequisite. Multicompartment models may also be used^22^ however single compartment models have by and large been sufficiently detailed to accurately predict voltages and currents^22–28^. The computational efficiency of single-compartment models is a significant advantage, particularly when processing large datasets. Moreover, single-compartment models are critical in conserving the observability of the neuron state and robustness against overfitting; they focus on capturing essential dynamics pertinent to the treatment response. We assume that while the electrophysiological data also include features such as transmission line delays, and the effects of spatial parameters and sensing domains, it is reasonable to consider these invariant to drug application, thus not influencing the predicted charge alterations in Figs. 3a, 4a, 5a, 6a. Absolute physiological realism of the model is not necessary, as we focus on the ability to detect alterations in reconstructed currents in response to a treatment, rather than absolute accuracy in the modelled ion channel responses. This approach provides the benefit of increasing DA speed and increasing the success rate of assimilations.

We now discuss the predicted channel block in relation to the subunit variation within each ion channel.

### Prediction of BK channel alterations

Our inference method correctly identifies a reduction in the BK-mediated current in response to application of IbTX which was the statistical discovery validated by the false discovery rate criterion (q < 1%) among all 7 ion channels analyzed (Figs. 3a,b). DA thus identifies correctly the effect of IbTX, a highly selective inhibitor of BK channels^37,38^. BK channels have a very high unitary conductance^39,40^, and contribute to both the repolarization of the action potential and to fast afterhyperpolarization (fI_AHP_), as seen in Fig. 3c. The contribution of the BK channel to both repolarization and fI_AHP_ were correctly predicted by the model, in addition to the reduction in overall BK current induced by IbTX (Fig. 3d). This is validation that DA transfers biological relevant information to the complete model.

Results predicted a 12.1% median reduction in the BK current (Table 1). The response of BK channels to IbTX is heavily modulated by the presence of up to four auxiliary subunits (β1-β4)^38,41,42^. Generally, β1 and β3 do not appear to be expressed in the brain. β2 is highly expressed in astrocytes, and β4 is expressed in neurons. It has been suggested that a full complement of four β subunits (1:1 stoichiometry) may be required to confer full IbTX resistance^43^: channels with less than four β subunits would exhibit toxin sensitivity similar to channels totally lacking β4 subunits^43^. As stoichiometry is unknown in these neurons, a mix of configurations would result in the partial inhibition of BK-mediated currents by IbTX. The potency of IbTX has previously been evaluated for several configurations of β subunits^44^ and is listed in Table 1. Whilst the precise degree of expected BK channel block is therefore not verifiable in CA1 neurons, we do find the channel is correctly selected by the method.

### Prediction of SK channel alterations

SK channels have a major role in generation of the AHP. Our predictions from recordings made using the highly specific SK channel blocker, apamin^45^, showed a median 100% reduction in SK-mediated current (Figs. 4a,b). The reduction in ranks for SK was the only statistical discovery, and DA did not predict any notable attenuation in charge transfer across any other type of ion channel. This validated the predictive power of our inference method against the specificity of inhibition by apamin.

Apamin is a highly selective inhibitor of SK2 and SK3 channels, which mediate medium AHP currents (mI_AHP_) with a relatively fast inactivation and decay^45^. Whilst apamin-sensitive I_AHP_ currents have been shown to be present in the CA1 soma, their blockade is often masked by the activity of other voltage-gated potassium channels^46,47^. The majority of SK channel subunits in CA1 neurons are SK2^48,49^, with SK3 showing relatively low expression. SK1 is expressed in moderate levels^50^ and is apamin insensitive, but does not contribute to mI_AHP_^51,52^. At 150 nM, apamin is expected to completely block SK2/3-mediated mI_AHP_^50^, and our prediction of an almost complete block (Fig. 4b) is therefore in excellent agreement with expected effects of apamin in CA1 neurons.

### Prediction of A-type and K channel alterations

4-aminopyridine was applied to inhibit voltage-dependent K^+^ channels. These are accounted for by the A-type and K-delayed rectifier channels in our model, each representing an amalgam of actual Kv channel subtypes, and we applied 4-AP to inhibit these two modelled channel groups simultaneously. A-type channels are known to be present in CA1 neurons^3^ where they give fast activating and fast inactivating K^+^ currents which can suppress excitatory postsynaptic potentials and delay action potentials^53^. In CA1 neurons, A-type K^+^ currents are mediated by either Kv1.4 or Kv4.2 channels^54^, with Kv4.2 being more abundant^55,56^. Our results show prediction of 24.3% median (19.0% mean) reduction in A-type K^+^ current (Figs. 5a,b), within the 13–25% inhibition expected from 4-AP at 300 μM (Table 1)^57,58^. This result is good evidence that the method is sensitive to smaller alterations in current.

The K-delayed-rectifier channels include the Kv1-3,5 and 6 subfamilies which are also inhibited by 4-AP, with an IC50 of 200–1500 µM^59,60^. We expected to see a reduction in predicted activity for the K-channel; however, whilst a reduction was clearly visible in 4-AP (Fig. 5a) this did not generate a discovery in the Mann–Whitney test. This is because the amount of charge transferred in the natural state is very low compared to other channels (NaT, A and BK). To statistically confirm the K-channel block, a larger sample of parameter estimates ($R \gg 19)$ would be needed to reduce the variance on the predicted charge transfer.

### Prediction of HCN channel alterations

Hyperpolarization-activated and cyclic nucleotide-gated (HCN) channels belong to the superfamily of voltage-gated pore loop channels. They are unique in possessing a reverse voltage-dependence that leads to activation upon hyperpolarization^61^. The HCN1 and HCN2 subunits are the most abundant in CA1 neurons^62–64^, and both are amalgamated in our model of the HCN channel. Under ZD7288, predicted HCN current was reduced by 100% median (85.5% mean) (Fig. 6a,b). This result is a good match to the degree of block expected from previous work on CA1 neurons indicating a 70–85% mean reduction in HCN-mediated current^65,66^. Similar values were obtained from specific studies of the HCN1^67^ and HCN2^68^ subunits (Table 1). In the predictive simulations of membrane voltage response to simulation (Fig. 6d), the average waveform of 19 iterations is presented. Notably, while the characteristic ‘sag’ of the HCN current is not prominently visible due to the averaging process, the ‘rebound’ phenomenon remains evident. An example of non-averaged predicted membrane voltage demonstrating the HCN ‘sag’ current is plotted in Fig. S4.

### Prediction of secondary effects of pharmacological inhibition in other channels

DA also predicted alterations in ion channels not specifically targeted by 4-AP and ZD7288 (Figs. 5a, 6a). The observation of collateral alterations is consistent with modification of the electrochemical driving force by the antagonist which alters current flow through other ion channels, particularly at times when the blocked channel would otherwise have been activated. For example, a reduction in K^+^ permeability during AHP will change the electrochemical driving force of other ions during that period. The driving force of Cl^-^ into the cell will increase whereas the Na^+^ driving force will be reduced. In addition, potassium current through the BK channel can compensate for the blocked Kv channels and vice-versa^69^.

The collateral effect of IbTX is to increase the leak current as predicted in Fig. 3a. This effect is likely to be caused by the reduction in K^+^ permeability when the large conductance BK channel is inhibited. It is also notable that within the 4-AP dataset (Fig. 5a), BK current increases when the A-type channel is blocked. This is a well characterized effect of 4-AP which causes a persistent K^+^ current^69^ and increases the spike width^70,71^ (Fig. 5c). Our model correctly predicts this spike broadening (Fig. 5d), and the DA method has sufficient sensitivity to pick up the second order increase in BK current (Fig. 5a). A small (4.4%) reduction in median Ca^2+^ channel current was also predicted in Fig. 5. This is likely to result from a reduction in the electrochemical driving force on Ca^2+^ caused by the decreased potassium permeability following each action potential.

The DA inference method has the potential to provide unbiased quantitative assessment of alterations among a range of ionic currents simultaneously, including current compensation between ion channels. The effects we observe are in good agreement with the selectivity and potency of antagonists, and notably we also detect well-characterized second order effects caused by blocking those channels. Sodium channels were not targeted in our study to avoid suppressing action potentials which would have impeded the ability of DA to estimate the parameters of all ion channels from the overall neuronal response. We decided therefore to prioritize other clinically relevant channels. However, future work would benefit from studying the partial inhibition of voltage gated Na channels.

The method we describe may be applied in various scenarios where assessing functional effects of drug or disease on specific ionic currents is desirable. Patch-clamp electrophysiology remains the primary technique used for profiling ion channels in vitro. A major limitation of this technique when applied traditionally is its low throughput, with a single ion channel being addressable at a time. This is especially limiting as a counter-toxicity screen when it is desirable to know the effects of a drug on more than one ionic current or when thousands of candidate drugs have to be screened. In drug screening, non-electrophysiological high throughput screening (HTS) methods such as ligand binding and ion flux assays can alternatively be applied. However, binding assays measure binding affinity rather than functional changes to ionic currents, and fluorescent assays are an indirect measure of such currents as well as being unsuitable for use with voltage-gated channels due to the lack of control over membrane voltage^72,73^. Ion flux assays are widely used in drug discovery, but also lack control over membrane voltage, as well as suffering from low temporal resolution and often weak ionic signal, rendering them inferior to voltage-clamp experiments^72^. Due to these limitations, the desire to apply patch-clamp assays early in the drug discovery process has led to the development of automated HTS patch-clamp systems^19^: such systems provide large amounts of functional data on the channels being targeted, however they are expensive to purchase and run, and like the above techniques, can normally only be applied in cell cultures directed to overexpress a specific ion channel rather than in primary tissue^73,74^. This renders them unable to infer the functional impact of candidate drugs in physiologically relevant systems such as acute primary tissue slices^19^. The DA method we present has the potential to be far faster than traditional patch clamp methods at interrogating multiple ionic currents at once, whilst retaining the ability to characterise the effects of a compound or treatment on individual neurons within a brain slice.

This proof-of-concept study may be applied to assay functional channel alterations in many other neuron types, in drug screening, and potentially in animal models of disease. When targeting other neurons, it may be necessary to add or remove channel groups from the model as appropriate based on information from prior studies. However, depending on the degree of overlap in the characteristics of modelled channel types, the addition of extraneous channels may not affect the accuracy of current reconstruction, as discussed previously. Further work will verify this in practice.

In relation to disease studies, the method could be complementary to transcriptomics and proteomics sequencing. Bottom-up sequencing methods do not discriminate between alterations which are relevant to electrical function from those which are not. Our top-down approach infers only the alterations in ion channels which are functionally relevant to neuronal electrical activity. Whilst the approach should be successful in theory, further validation of the method using animal models of channelopathy are necessary.

In summary, the present study demonstrates that it is possible to reliably reconstruct multiple specific ionic currents by assimilating the membrane voltage of a neuron driven by a complex current waveform. Accuracy on the reconstructed ionic currents is sufficient to predict alterations in currents in agreement with the expected effects of inhibitory compounds, as well as predicting well-characterized second order compensation effects. This data assimilation method requires no prior assumption as to which channel might be affected as it provides a quantitative assessment of functional alterations among a range of ionic currents in one shot, which to our knowledge has not previously been achieved. With further validation it therefore has the potential to be widely applied in drug screening pipelines, and additionally in studies aiming to characterize ion channel dysfunction in disease models. It has the benefit of application in acute tissue slices or primary neuron cultures and may substantially reduce workload.

## Materials and methods

### Current clamp electrophysiology

CA1 hippocampal neurons were driven and recorded using a Molecular devices MultiClamp 700B amplifier. This type of amplifier uses a voltage follower circuit that was necessary to drive rapidly varying currents. A LabView controller (National Instruments) interfaced with a National Instruments USB-6363 DAQ card delivered the clamp protocol signal to the amplifier and recorded the membrane voltage returned by the neuron. Prior to each series of experiments, the gain of the protocol (via a multiplier) was adjusted to elicit a maximum number of action potentials per measurement epoch without causing depolarization block with excessive current amplitudes. The calibration protocol is described in Fig. S2. Current clamp protocols were designed to fulfil the identifiability criterion of the inverse problem, that is to excite the full dynamic range of the neuron. It comprised a mixture of hyperpolarizing and depolarizing current steps of different amplitudes and durations, and chaotic oscillations generated by the Lorenz96 system. Both the current stimulus and the membrane voltage were sampled at a rate of 100 kHz. This time resolution gave 20 datapoints per action potential which is sufficient for interpolating the finer features of the neuron response.

Whole-cell current-clamp recordings were performed in acute brain slices from male Han Wistar rats at P15–17. Following decapitation, the brain was removed and placed into an ice-cold slicing solution composed of (mM): NaCl 52.5; sucrose 100; glucose 25; NaHCO_3_ 25; KCl 2.5; CaCl_2_ 1; MgSO_4_ 5; NaH_2_PO_4_ 1.25; kynurenic acid 0.1, and carbogenated using 95% O_2_/5% CO_2_. A Campden 7000 smz tissue slicer (Campden Instruments UK) was used to prepare transverse hippocampal slices at 350 μm, which were then transferred to a submersion chamber containing carbogenated artificial cerebrospinal fluid (aCSF) composed of (mM): NaCl 124; glucose 30; NaHCO_3_ 25; KCl 3; CaCl_2_ 2; MgSO_4_ 1; NaH_2_PO_4_ 0.4 and incubated at 30 °C for 1–5 h prior to use. Synaptic transmission was inhibited pharmacologically in order to prevent network feedback or random postsynaptic potentials from disrupting the trace. To this end all experiments were performed in the presence of (μM) kynurenate 3, picrotoxin 0.05, and strychnine 0.01, to inhibit ionotropic glutamatergic, γ-aminobutyric acid (GABA)-ergic, and glycinergic neurotransmission respectively.

For patching, slices were transferred to the stage of an Axioskop 2 upright microscope (Carl Zeiss) and pyramidal CA1 neurons identified morphologically and by location using differential interference contrast optics. The chamber was perfused with carbogenated aCSF (composition as above) at 2 ml min^−1^ at 30 ± 1 °C. Patch pipettes were pulled from standard walled borosilicate glass (GC150F, Warner Instruments) to a resistance of 2.5–4 MΩ, and filled with an intracellular solution composed of (mM): potassium gluconate 130; sodium gluconate 5, HEPES 10; CaCl_2_ 1.5; sodium phosphocreatine 4; Mg-ATP 4; Na-GTP 0.3; pH 7.3; filtered at 0.2 µm.

Inhibitory compounds were selected for the predictability of their effects on ion channel types known to be present in hippocampal pyramidal neurons:

- SK channels were inhibited using apamin (150 nM);
- BK channels were inhibited with iberiotoxin (100 nM);
- HCN channels were inhibited with ZD7288 (50 µM);
- A and K channels were inhibited using 4-AP (300 µM).

The potency of each drug was obtained from IC50 values tabulated in the literature (Table 1), which we compared to the reduction in ionic charge transfer predicted by our DA method. A total of 13 animals were used in development of the methodology. The data presented in this proof-of-concept study were collected from 4 animals. Each current datapoint was computed from one assimilation window, from a single neuron, in the pre-drug or the post-drug state using a single compound as specified. The chaotic current clamp protocol was applied pre-drug, before immediately switching to drug-containing aCSF at the specified concentration and allowing to wash in for a further 3 min, before initiation of the same protocol. Time between the start of the pre-drug clamp protocol and termination of the drug-applied protocol was in every case < 5 min.

### Model description

A single-compartment model of the CA1 pyramidal neurons was built using a conductance-based framework incorporating eight active ionic currents identified in the physiological literature as being prevalent in the soma of CA1 neurons^62,66,75^, in addition to a voltage-independent leak current^76,77^. The complement of ionic channels includes transient sodium (NaT), persistent sodium (NaP), delayed-rectifier potassium (K), A-type potassium (A), low threshold calcium (Ca), large- and small-conductance Ca^2+^- activated potassium (BK and SK respectively), and the hyperpolarization-activated cation channel (HCN). The density of calcium channels in the soma of CA1 neurons is much lower than in distal dendrites^78^, however the internal Ca^2+^ concentration activates the transfer of K^+^ ions through the Ca-dependent BK and SK channels. Therefore our model equations need to include the calcium current. The equation of motion for the membrane voltage is:

1 $$C\frac{dV\left( t \right)}{{dt}} = - J_{NaT} - J_{NaP} - J_{K} - J_{A} - J_{Ca} - J_{BK} - J_{SK} - J_{HCN} - J_{Leak} + I_{inj} \left( t \right)/A,$$

where *C* is the membrane capacitance, *V* is the membrane potential, *I*_inj_(t) is the injected current protocol (Fig. 1a), *A* is the surface area of the soma, and *J*_NaT_ … *J*_Leak_ are the ionic current densities across the cell membrane. The equations describing individual ionic currents are given in Table S1. These currents depend on maximum ionic conductances (*g*_NaT_, *g*_K_, *g*_HCN_…), reversal potentials (*E*_Na_, *E*_K_, *E*_HCN_…), and gating variables (*m*, *h*, *n*, *p*, …). The kinetics of each ionic gate is described by a first order equation and each gate activates or inactivates according to a sigmoidal function of the membrane voltage. The equations for each ion channel are as follows:

#### Sodium channels

The *activation* gate variables of the NaT and NaP channels were respectively:

2 $$m_{\infty } \left( V \right) = 0.5\left[ {1 + \tanh \left( {\frac{{V - V_{m} }}{{\delta V_{m} }}} \right)} \right],$$

3 $$p_{\infty } \left( V \right) = 0.5\left[ {1 + \tanh \left( {\frac{{V - V_{p} }}{{\delta V_{p} }}} \right) } \right],$$

where $V_{m}$, $V_{p}$ are the activation thresholds and $\delta V_{m} , \delta V_{p}$ are the widths of the gate transition from the open to the closed state. The activation time of NaT and NaP being very rapid (~ 0.1 ms)^73^ compared to other channels we have assumed it to be instantaneous. This simplification reduces model complexity and improves parameter identifiability in DA.

The kinetics of the NaT *inactivation* gate is given by:

4 $$\frac{{dh\left( {V,t} \right)}}{dt} = \frac{{h_{\infty } \left( V \right) - h\left( {V,t} \right)}}{{\tau_{h} \left( V \right)}},$$

where the steady-state inactivation curve is:

5 $$h_{\infty } \left( V \right) = 0.5\left[ {1 + \tanh \left( {\frac{{V - V_{h} }}{{\delta V_{h} }}} \right)} \right],$$

and the recovery time depends on the membrane voltage as:

6 $$\tau_{h} \left( V \right) = t_{h} + \epsilon_{h} \left[ {1 - \tanh^{2} \left( {\frac{{V - V_{h} }}{{\delta V_{\tau h} }}} \right) } \right].$$

$V_{h}$ is the inactivation threshold, $\delta V_{h}$ the width of the open-to-closed transition of the inactivation gate. $t_{h}$ is recovery time away from the depolarization threshold and $t_{h} + \epsilon_{h}$ the recovery time at the depolarization threshold. $\delta V_{\tau h}$ is the width of the peak at half maximum.

#### Potassium channels

The non-inactivating delayed-rectifier current (K) and the rapidly inactivating A-type potassium current (A) have the form^77^ given in Table S1. The kinetics of the A-type activation gate is:

7 $$\frac{{da\left( {V,t} \right)}}{dt} = \frac{{a_{\infty } \left( V \right) - a\left( {V,t} \right)}}{{\tau_{a} \left( V \right)}},$$

where $a_{\infty } \left( V \right)$ and $\tau_{a} \left( V \right)$ are given by Eqs. 5, 6 where the subscript *h* is replaced with *a* (Table S2).

The inactivation kinetics of the K and A-type channels are respectively given by:

8 $$\frac{{dn\left( {V,t} \right)}}{dt} = \frac{{n_{\infty } \left( V \right) - n\left( {V,t} \right)}}{{\tau_{n} \left( V \right)}},$$

9 $$\frac{{db\left( {V,t} \right)}}{dt} = \frac{{b_{\infty } \left( V \right) - b\left( {V,t} \right)}}{{\tau_{b} \left( V \right)}},$$

where $n_{\infty } \left( V \right)$, $\tau_{n} \left( V \right)$ and $b_{\infty } \left( V \right)$ and $\tau_{b} \left( V \right)$ are given by Eqs. 5, 6 with the appropriate substitution of indices (Table S2). Although the muscarinic potassium current ($I_{M}$) is present in certain CA1 neurons, it was excluded from our model because of its relatively minor conductance and its persistent activity^78^ which primarily modulates the resting potential of the CA1 neuron^79^. We determined that the characteristics of the $I_{M}$ can be adequately captured by the parameters governing the A-type potassium current, thereby avoiding an unnecessary increase in model complexity.

#### Calcium activated potassium channels

The BK and SK currents are Ca^2+^ activated potassium currents found in the soma of hippocampal pyramidal cells^78^. The BK current is sensitive to both membrane voltage and internal Ca^2+^ concentration whereas the SK current only depends on the Ca^2+^ concentration (Table S1). Both currents are dependent of the internal calcium concentration given by^80^:

10 $$\frac{{d\left[ {Ca} \right]_{in} }}{dt} = \frac{{\left[ {Ca} \right]_{\infty } - \left[ {Ca} \right]_{in} }}{{\tau_{ca} }} - \frac{{J_{Ca} }}{2wz} ,$$

where $\left[ {Ca} \right]_{\infty }$ is the equilibrium concentration, $\tau_{ca}$ is t he recovery time, *z* is Faraday’s constant, $w$ is the thickness of the surface across which Ca^2+^ fluxes are calculated ($w = 1\mu m$), and $J_{ca}$ is the calcium current whose expression is given in Table S1. The calcium current had voltage-dependent activation and inactivation gates, *s* and *r*, respectively^80^. The kinetics and activation curves of *s* and *r* are given by Eqs.4–6 where subscript *h* is replaced with the *s* and *r* subscripts of the Ca parameters (Table S2). The relaxation time constant for Ca^2+^ in our model is set between 1 and 2 ms, a range that was chosen based on the slow dynamics, characteristic of Ca^2+^ signaling. In the context of the model equations governing calcium, small variations within this interval were found to substantially influence the rate of change of internal Ca^2+^ concentration, and so this range was deemed sufficient to accurately reflect the diversity of calcium dynamics in CA1 neurons.

The BK current has two gate variables *c*, *d* while the SK channel has one *w*. The form of the ultrafast SK activation gate, *w*, is given by Warman et al.^81^ as:

11 $$w \equiv w_{\infty } \left( {\left[ {Ca} \right]_{in} } \right) = 0.5\left[ {1 + \tanh \left\{ {\left\{ {V - V_{w} + 130\left\{ {1 + \tanh \left( {\frac{{\left[ {Ca} \right]_{in} }}{0.2}} \right) } \right\} - 250} \right\}/\delta V_{w} } \right\} } \right]$$

The slower activation gate of the BK channel, c, follows a first order rate equation:

12 $$\frac{d c}{{dt}} = \frac{{c_{\infty } \left( {V,\left[ {Ca} \right]_{in} } \right) - c}}{{\tau_{c} }}$$

with a steady-state activation curve given by:

13 $$c_{\infty } \left( {V,\left[ {Ca} \right]_{in} } \right) = 0.5\left[ {1 + \tanh \left\{ {\left\{ {V - V_{c} + 130\left\{ {1 + \tanh \left( {\frac{{\left[ {Ca} \right]_{in} }}{0.2}} \right) } \right\} - 250} \right\}/\delta V_{c} } \right\} } \right]$$

The inactivation gate of the BK channel, d, similarly follows a first order rate equation:

14 $$\frac{d d}{{dt}} = \frac{{d_{\infty } \left( {V,\left[ {Ca} \right]_{in} } \right) - d}}{{\tau_{d} \left( V \right)}}$$

with

15 $$d_{\infty } \left( {V,\left[ {Ca} \right]_{in} } \right) = 0.5\left[ {1 + \tanh \left\{ {\left\{ {V - V_{d} + 130\left\{ {1 + \tanh \left( {\frac{{\left[ {Ca} \right]_{in} }}{0.2}} \right) } \right\} - 250} \right\}/\delta V_{d} } \right\} } \right]$$

16 $$\tau_{d} \left( V \right) = t_{d} + \epsilon_{d} \left[ {1 - \tanh^{2} \left( {\frac{{V - V_{d} }}{{\delta V_{\tau d} }}} \right) } \right]$$

The existence of the SK and BK ionic currents was validated by much improved fits of the height and shape of action potentials, and their AHP region (Fig. 1e). Without the SK and BK currents, the model clips action potentials at 80% of their maximum height. In total, our conductance model had the 67 adjustment parameters listed in Table S2.

### Parameter estimation and current prediction

Our interior point method optimizes the parameter vector ${\varvec{p}}^{\user2{*}}$ and the initial state vector x*(*t* = 0) by minimizing the misfit between the experimental membrane voltage, *V*_data_, and the membrane voltage variable, *V*, at each time point $t_{i}$ ($i = 0, \ldots ,N$) of the assimilation window (Fig. 1a). This misfit is evaluated by the least-square cost function:

17 $$C\left( {p, {\varvec{x}}\left( 0 \right)} \right) = \frac{1}{2}\mathop \sum \limits_{i = 0}^{N} \left\{ {\left[ {V_{data} \left( {t_{i} } \right) - V\left( {t_{i} , {\varvec{p}},\user2{ x}\left( 0 \right)} \right)} \right]^{2} + u\left( {t_{i} } \right)^{2} } \right\}.$$

The cost function is minimized under both equality and inequality constraints using the variational approach of Lagrangian optimization. The equality constraints are the model equations (Eqs.1–16). These were linearized at each time point $t_{i}$ of the assimilation window^21,30^ which was 800 ms long and was meshed by *N* = 40,000 intervals of equal duration. The inequality constraints are given by the lower and upper boundaries of the parameter search range, LB and UB, in Table S2. These are set by the user. The 67 parameter components of the parameter vector ${\varvec{p}}^{\user2{*}}$ are listed in Table S2. The state vector has 14 state variables, ${\varvec{x}}\left( t \right) \equiv \left\{ {V\left( t \right),m\left( t \right), h\left( t \right), p\left( t \right), n\left( t \right), a\left( t \right), b\left( t \right), s\left( t \right), r\left( t \right),c\left( t \right), d\left( t \right), w\left( t \right),z\left( t \right),\left[ {Ca} \right]_{in} } \right\}$ that hold the membrane voltage, gate variables, and internal calcium concentration. State variable $V\left( t \right)$ is observed, and is synchronized to the data, whereas the other state variables $m\left( t \right), \ldots ,\left[ {Ca} \right]_{in}$ are unobserved and must be inferred. We used symbolic differentiation within Python to compute the Jacobian of the state variables with respect to parameters and the Hessian of the cost function. Both matrices were then inserted in the interior-point-optimization algorithm developed by Wächter and Biegler^29^ that iteratively determines ${\varvec{p}}^{\user2{*}}$ and x*(*t*_*i*_) at each point of the assimilation window. Thus, data assimilation infers observed and unobserved state variables, and model parameters. It estimates both parameters that relate in a nonlinear way to the membrane voltage (gate voltage thresholds, activation slopes, gate recovery times) as well as linear parameters (ionic conductances).

In order to stabilize the convergence of the parameter search, a control term *u*(*t*_i_)[*V*_data_(*t*_i_*)-V*(*t*_i_)] was added to the right-hand side of Eq. 1^21,82^ and as *u*^2^(*t*_i_) in Eq. 17. In well-posed assimilation problems, the Tikhonov regularization term^82^ *u*(*t*_0_) …* u*(*t*_N_) uniformly tends to zero as ***p*** converges to the solution p^*^. The model error and experimental error encountered with biological neurons makes the problem ill-posed. Model error introduces correlations between some parameters which take multi-valued solutions when the initial guesses on state variables, parameters or data intervals vary. In this case the $u\left( {t_{i} } \right)$ also converge to zero except at times that coincide with action potentials. Models configured with optimal parameters that include a small subset of correlated parameters reliably predict membrane voltage oscillations and ionic current waveforms for a wide range of current injection protocols (Figs. 1, S1). When the *u*(*t*_i_) failed to converge uniformly across the assimilation window, the estimated parameters were discarded from the statistical analysis of the ion channels. Models configured with such parameters were unable to predict the experimental membrane voltage, as for example in Fig. 1e. Prior to the analysis of biological recordings, we verified that our current protocol and DA procedure fulfilled the conditions of observability and identifiability on model data. These preliminary studies showed that DA recovered *all* 67 parameters to within 0.1% of their original value in the model used to produce the assimilated data. We verified the uniqueness and accuracy of solutions using the *R* = 19 assimilation windows offset by 80 ms (Fig. 1a) and varying the starting values of ${\varvec{p}}^{\user2{*}}$ and ${\varvec{x}}^{\user2{*}}$.

The predicted ionic currents and membrane voltages were generated by forward integration of each completed model over the 2000 ms long epoch, both pre- and post-drug. Current waveforms were integrated to obtain the total charge transferred through each channel in that epoch (Figs. 3e, 4e, 5e). In order to eliminate the dependence on the neuron firing frequency, we divided the total charge transferred across the epoch by the number of action potentials to obtain the net charge transferred per spike, per ion channel (Figs. 3a, 4a, 5a). To verify the predicted inhibition is not affected by integrating currents over one action potential rather than the entire assimilation window, we plotted the changes in *total* charge transferred over the full epoch both pre- and post-drug (Fig. S3). We verify that both methods gave similar results with small differences arising from sub-threshold current flow between action potentials.

The model equations were differentiated symbolically using our custom-built Python library pyDSI to generate the C++ code of the optimization problem^22–24^. This code was then inserted in the open-source IPOPT software [www.coin-or-org/ipopt] implementing the MA97 sparse linear equation solver [http://www.hsl.rl.ac.uk/catalogue]. The optimizations were run on a 16-core (3.20 GHz) Linux workstation with 64 GB of RAM and a University of Bath minicomputer with 64-core processors and 320 GB of RAM. Model equations were linearized according to Boole’s rule^30^.

### Statistical analysis

Extreme outliers in the predicted charge data were detected using the ROUT test^83^ with the maximum desired false discovery rate, Q set at 0.1%, based on values for the NaT channel. Only 3 outliers were identified out of a total of 138. The corresponding parameters solutions $p^{*}$ could also be identified by their failure to predict the membrane voltage oscillations over the 2000 ms epoch.

Due to the non-gaussian distributions of some of the total predicted charge data, multiple two-tailed Mann–Whitney U^84^ rank-sum tests were applied, with multiple comparisons corrected for using the two-stage step-up method of Benjamini, Krieger and Yekutieli, with Q at 1%. Mann–Whitney U values are reported, and multiplicity-corrected significance values (q) are therefore reported for all discoveries. In figures, asterisks are applied based on these q values. For comparisons where predicted charge transfer distributions differed pre-drug and post-drug, we report the mean rank values in relation to Mann–Whitney U test output. GraphPad Prism version 9 was used for all statistical analyses.

### Ethical statement

Experiments on rodents were performed under Schedule 1 in accordance with the United Kingdom Scientific Procedures act of 1986.

### Supplementary Information

Supplementary Information.

## Data Availability

Electrophysiological recordings underpinning this study are archived on the open data base https://researchdata.bath.ac.uk.
