# Supplementary material for: Single shot detection of alterations across multiple ionic currents from assimilation of cell membrane dynamics
Source: Sci Rep. 2024 Mar 12;14:6031. doi: 10.1038/s41598-024-56576-3 (PMC10933487; doi:10.1038/s41598-024-56576-3)
Supplement: Supplementary file 1 — Supplementary Information. [file 41598_2024_56576_MOESM1_ESM.docx]

Supplementary Materials for

**Single shot detection of alterations across multiple ionic currents from assimilation of cell membrane dynamics**

Paul G. Morris, Joseph D. Taylor, Julian F.R. Paton, Alain Nogaret*

*Corresponding author. Email: A.R.Nogaret@bath.ac.uk

**This PDF file includes:**

Supplementary Text

Tables S1 to S2

Figures S1 to S4

**Other Supplementary Materials for this manuscript include the following:**

Source data file

**Supplementary text: Computational method and analysis of data**

1. **Completed models successfully predict membrane oscillations over 2s long epochs**

**Figure S1** shows the predictions of the completed model over the full 2000ms long epoch. This epoch covers a wider time interval than the few action potentials of Figures.3-5. The membrane voltage predicted by the pre-drug model is compared to the measured membrane voltage time (Panel A) and similarly, the membrane voltage predicted by the post-drug model is compared to the membrane voltage measured after applying apamin (panel B). In each case, the models make excellent predictions of the membrane voltage over the 2000ms long epoch. Details are shown in panels C and D.

1. **Calibrating the amplitude of the stimulating current**

In order to determine the range of optimal current stimulation prior to any measurement being taken, we conducted the calibration exercise depicted in **Figure S2a**. This protocol injected 50ms long current steps increasing gradually from 20pA to a maximum of 600pA in 20pA increments. The optimal level of current stimulation maximizes the number of action potentials per epoch while minimizing depolarization block that occurs at higher stimulation. In this way, a maximum amount of information could be transferred from the biological data to the model. Once the optimal level of current stimulation was established, we applied the appropriate scaling factor to each of the 150 protocols generating the assimilation data. **Figure S2b** is an example of a suitably scaled current protocol in contrast to **Figure S2c** which has fewer action potentials when stimulation is too large (depolarization block). Low neuron firing rates occur both at the low end of the stimulation range when the current is near the threshold and at the high end when the neuron undergoes depolarization block.

1. **Ionic charge integrated over the 2s long epoch**

Here we present an alternative method for calculating the charge transfer per ion channel. Instead of calculating the charge transferred per action potential as done in Figures 3-5, we calculate the charge transferred across the entire 2000ms assimilation window through both sub-threshold regions and action potentials (**Figure S3**). Ionic charge transfer during sub-threshold oscillations is expected to be negligible, however this needs to be verified to demonstrate the robustness of the claimed predictions and their independence on the method used. The percentage drop in ionic charge transfer across the 2000ms window is quoted in dark bold **[…%]** against the percentage drop per action potential in green bold font **[…%]**.

*BK channel blockade*

The BK channel blocker, iberiotoxin (IbTX; 100 nM; pre-drug assimilations/predictions *N*=15; drug applied=15) reduced the transfer of BK-specific charge by **13.1% [12.1%]** (U=28.5; q<0.01) relative to the pre-drug state. The median charge transfer across the 2000ms window was 1.697 µC.cm^-2^ pre-drug, and 1.474 µC/cm^-2^ in IbTX (**Figure S3 A, B**). Data assimilation also predicted an increase in leak current in IbTX (U=41.5; q<0.01; mean ranks 10.8 [pre-drug], 20.2 [IbTX]). This is a secondary effect caused by the reduction in K^+^ permeability when inhibiting K^+^ channels, increasing the driving force of Cl^-^ into the cell. The mean number of spikes over the 2000ms window was 39.47 ± 0.86 before IbTX, and 38.40 ± 0.855 after IbTX was applied (*N* values as above).

*SK channel blockade*

The SK-specific channel blocker, apamin (150 nM; pre-drug assimilations/predictions *N*=18; drug applied=18), reduced the transfer of SK-specific charge (U=68; q<0.01; mean ranks 23.7 [pre-drug], 13.3 [apamin]). Median SK charge transfer was by **100%** **[100%]** lower relative to the pre-drug state (**Figure S3 C, D**). Data assimilation predicted an additional increase in charge transfer through the voltage-gated Na^+^ channel (U=56; q<0.01; mean ranks 12.6 [pre-drug], 24.4 [apamin]) and the A-type K^+^ channel (U=66; q<0.01; mean ranks 13.2 [pre-drug], 23.8 [apamin]).

*Blockade of A-type channels*

The application of 300 µM of the Kv channel blocker 4-AP (pre-drug assimilations/predictions *N*=19; drug applied=18), reduced the transfer of A-type specific charge (U=62; q<0.001; mean ranks 24.7 [pre-drug], 12.9 [4-AP]). Median A-type specific charges was by **15.5%** **[19%]** lower relative to the pre-drug state (**Figure S3 E, F**). Data assimilation also predicted an increase in charge transfer through the BK (U=40; q<0.0001; mean ranks 12.1 [pre-drug], 26.3 [4-AP]), voltage-gated Na^+^ (U=59; q<0.001; mean ranks 13.1 [pre-drug], 25.2 [4-AP]), and Ca^2+^ (U=83; q=0.01; mean ranks 14.4 [pre-drug], 23.9 [4-AP]) channels.

In summary computing the charge transferred per action potential per ion channel (BK, SK and A-type) gives very similar results to computing the charge transferred per ion channel across the entire assimilation window.

| **ID** | **Channel** | **Current density** |
| --- | --- | --- |
| NaT | Transient sodium current | $J_{NaT}=g_{NaT} m_{\infty} h^{3} (E_{Na}-V)$ |
| NaP | Persistent sodium current | $J_{NaP}=g_{NaP} p_{\infty}(E_{Na}-V)$ |
| K | Delayed-rectifier potassium current | $J_{K}=g_{K}n^{4}(E_{K}-V)$ |
| A | A-type potassium current | $J_{A}=g_{A} a b (E_{K}-V)$ |
| Ca | Calcium current | $J_{Ca}=g_{Ca} s^{2} r (E_{Ca}-V)$ |
| BK | Large conductance calcium-activated potassium current | $J_{BK}=g_{BK} c^{2} d (E_{K}-V)$ |
| SK | Small conductance calcium-activated potassium current | $J_{SK}=g_{SK} w (E_{K}-V)$ |
| HCN | Hyperpolarization-activated cation current | $J_{HCN}=g_{H} z (E_{HCN}-V)$ |
| Leak | Leakage current | $J_{Leak}=g_{L}(E_{L}-V)$ |

**Table S1. The nine ionic currents of our hippocampal neuron model.**

The voltage dependence of the ionic current densities is shown in the right column. Parameters include ionic conductances $g_{i=\{NaT, NaP, K, A, Ca, BK, SK, HCN, L\}}$, reversal potentials $E_{j=\{Na, K, Ca, HCN, L\}}$ listed in Table S2. The model has 14 gate variables $\{V,m, h,p, n, a, b, s, r, c,d, w,z,\left[ Ca \right]_{in}\}$ and 67 parameters.

| $\boldsymbol{i}$ | $\boldsymbol{p}_{\boldsymbol{i}}$ | | **Units** | **LB** | **UB** |
| --- | --- | --- | --- | --- | --- |
| 1 | Cap. | C_m_ | μF/cm^2^ | 1 | 1 |
| $2$ | NaT | $g_{NaT}$ | nS/cm^2^ | 5 | 100 |
| 3 | NaP | $g_{NaP}$ | nS/cm^2^ | 5 | 100 |
| 4 | Na | *E_Na_* | mV | 60 | 70 |
| 5 | K | $g_{K}$ | nS/cm^2^ | 5 | 20 |
| 6 | HCN | $g_{HCN}$ | nS/cm^2^ | 0 | 0.3 |
| 7 | K | *E_K_* | mV | -110 | -90 |
| 8 | Leak | *E_L_* | mV | -75 | -55 |
| 9 | HCN | *E_HCN_* | mV | -60 | -40 |
| 10 | Leak | $g_{L}$ | nS/cm^2^ | 0.2 | 1 |
| 11 | NaT | *V_m_* | mV | -40 | -25 |
| 12 | NaT | *δV_m_* | mV | 5 | 20 |
| 13 | NaT | *V_h_* | mV | -70 | -50 |
| 14 | NaT | *δV_h_* | mV | -30 | -5 |
| 15 | NaT | *δV_τh_* | mV | 20 | 40 |
| 16 | NaT | *t_h_* | ms | 0.1 | 2.0 |
| 17 | NaT | *ε_h_* | ms | 5 | 20 |
| 18 | NaP | *V_p_* | mV | -40 | -25 |
| 19 | NaP | *δV_p_* | mV | 5 | 20 |
| 20 | K | *V_n_* | mV | -40 | -25 |
| 21 | K | *δV_n_* | mV | 5 | 25 |
| 22 | K | *δV_τn_* | mV | 5 | 25 |
| 23 | K | *t_n_* | ms | 0.1 | 2.0 |
| 24 | K | *ε_n_* | Ms | 1 | 10 |
| 25 | A | $g_{A}$ | nS/cm^2^ | 1 | 100 |
| 26 | A | *V_a_* | mV | -20 | 5 |
| 27 | A | *δV_a_* | mV | 5 | 25 |
| 28 | A | *δV_τa_* | mV | 5 | 25 |
| 29 | A | *t_a_* | ms | 0.1 | 2.0 |
| 30 | A | *ε_a_* | ms | 1.0 | 20 |
| 31 | A | *V_b_* | mV | -90 | -80 |
| 32 | A | *δV_b_* | mV | -20 | -5 |
| 33 | A | *δV_τb_* | mV | 20 | 30 |
| 34 | A | *t_b_* | ms | 5 | 50 |

| $\boldsymbol{i}$ | $\boldsymbol{p}_{\boldsymbol{i}}$ | | **Units** | **LB** | **UB** |
| --- | --- | --- | --- | --- | --- |
| 35 | A | *ε_b_* | Ms | 5 | 50 |
| 36 | Ca | $g_{Ca}$ | nS/cm^2^ | 9 | 12 |
| 37 | Ca | *E_Ca_* | mV | 120 | 120 |
| 38 | Ca | *V_s_* | mV | -35 | -25 |
| 39 | Ca | *δV_s_* | mV | 10 | 20 |
| 40 | Ca | *δV_τs_* | mV | 30 | 40 |
| 41 | Ca | *t_s_* | Ms | 0.01 | 0.1 |
| 42 | Ca | *ε_s_* | Ms | 0.1 | 2 |
| 43 | Ca | *V_r_* | mV | -70 | -55 |
| 44 | Ca | *δV_r_* | mV | -20 | -10 |
| 45 | Ca | *δV_τr_* | mV | 20 | 30 |
| 46 | Ca | *t_r_* | Ms | 0.1 | 1.0 |
| 47 | Ca | *ε_r_* | Ms | 1.0 | 10 |
| 48 | BK | $g_{BK}$ | nS/cm^2^ | 0 | 100 |
| 49 | BK | *V_c_* | mV | -20 | -10 |
| 50 | BK | *δV_c_* | mV | 5 | 30 |
| 51 | BK | τ*_c_* | Ms | 1.1 | 1.1 |
| 52 | BK | *V_d_* | mV | -60 | -40 |
| 53 | BK | *δV_d_* | mV | -20 | -5 |
| 54 | BK | *δV_τd_* | mV | 5 | 30 |
| 55 | BK | *t_d_* | Ms | 0.1 | 2.0 |
| 56 | BK | *ε_d_* | Ms | 1.0 | 20 |
| 57 | SK | $g_{SK}$ | nS/cm^2^ | 0 | 0.05 |
| 58 | SK | *V_w_* | mV | 0.5 | 1.0 |
| 59 | SK | *δV_w_* | mV | 0.3 | 0.5 |
| 60 | HCN | *V_z_* | mV | -90 | -70 |
| 61 | HCN | *δV_z_* | mV | -20 | -1 |
| 62 | HCN | *δV_τz_* | mV | 10 | 30 |
| 63 | HCN | *t_z_* | Ms | 1 | 10 |
| 64 | HCN | *ε_z_* | Ms | 10 | 200 |
| 65 | *Area* | *A* | x10^4^ μm^2^ | 1.0 | 5.0 |
| 66 | Ca | *τ_Ca_* | Ms | 1.0 | 2.0 |
| 67 | Ca | [Ca]_∞_ | mM | 0.001 | 0.001 |

**Table S2. List of model parameters with their search interval [LB-UB]**

The model parameters and parameter search intervals for the conductance-based model used for assimilating hippocampal neuron data. LB and UB are the lower and upper boundaries of the parameter search interval respectively.


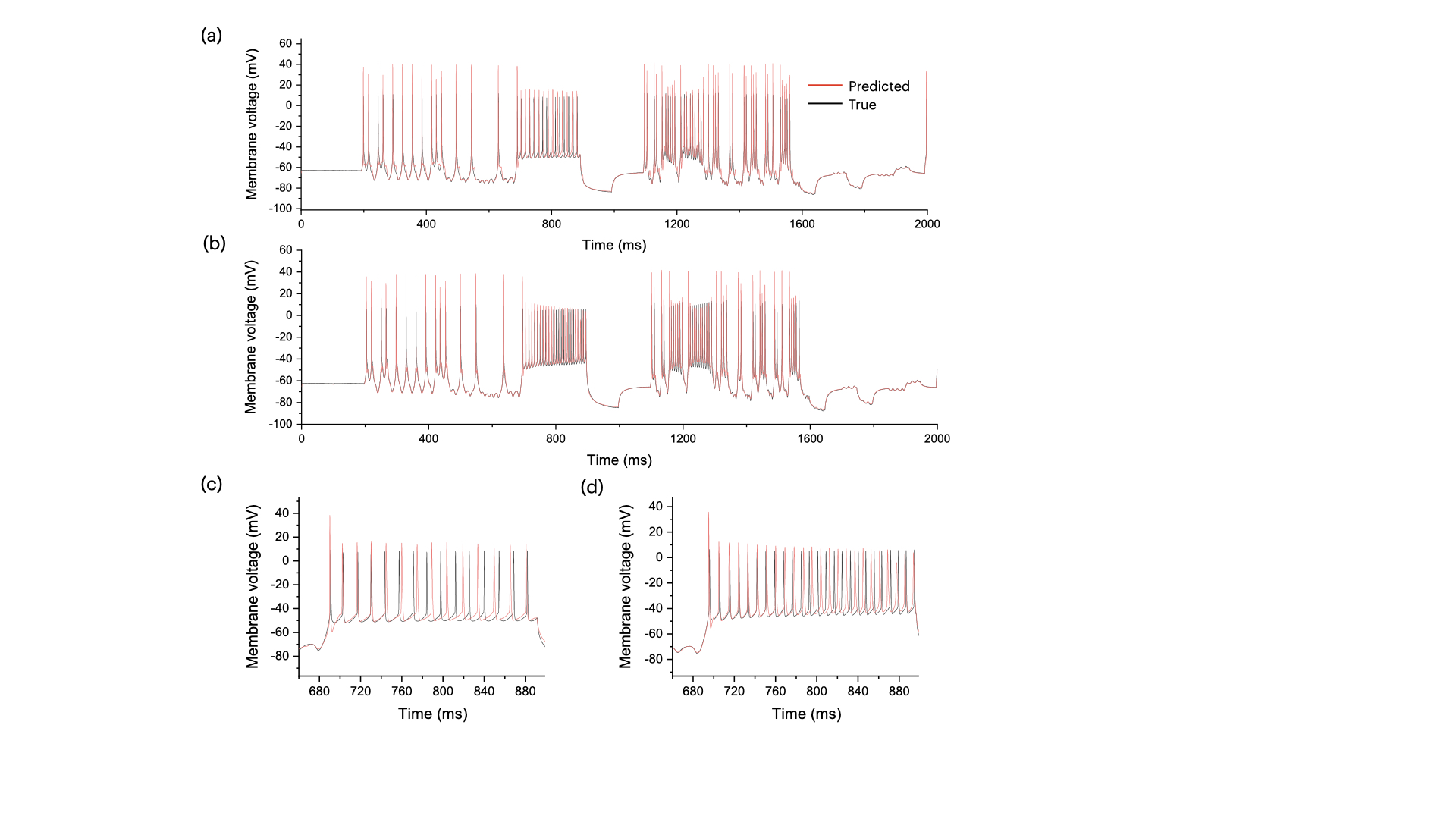


Predicted

Experiment

**A** We investigated parameters extracted from model data incorporating both model error (an erroneous gate exponent) and noisy model data. **Figure S2a** shows that some parameters were more loosely constrained than others. The covariance matrix of the parameter field shows correlations between parameters which occur mainly within blocks (**Figure S2b**). Each block of parameters corresponds precisely to the parameters defining a single ionic current. The spectrum of eigenvalues of the covariance matrix (**Figure S2c**) showed 4 longer semi-axes along which parameter correlations are the greatest. Other eigenvalues decay rapidly to zero (faster than exponentially) showing that most parameters remain well constrained (**Figure S2a**). We reconstructed the ionic currents from these parameter estimates and compared the variance on currents to the variance on parameters (**Figure S2d**). The results show a 3 times higher accuracy on the reconstructed currents than on the parameter themselves. This is due to the uncertainty on parameters arising from correlations rather than randomness. These correlations cancel out to a large extent by integration when reconstructing each ionic current. This is the property that claims the identification of the blocked ion channels from our predictions.

**C**

**B**

**Figure S1. Long-term prediction of membrane voltage by the completed model before and after the application of apamin (SK blockade)**.

(**A**) 2000-ms-long epoch showing the membrane voltage measured from a CA1 hippocampal neuron (black line) and the predicted membrane voltage (red line). The predicted trace was obtained by forward integrating the completed CA1 neuron model stimulated by the same current protocol as the real neuron. (**B**) 2000-ms-long epoch showing the same neuron after application of SK-channel blocker apamin (150 nM). The predicted membrane voltage was computed from a new completed model. (**C, D**) Detail of the membrane voltage response to the step current at t=680ms pre- and post-channel blockade, respectively. The model successfully replicates the increase in firing frequency after the blocking of the SK potassium channel.


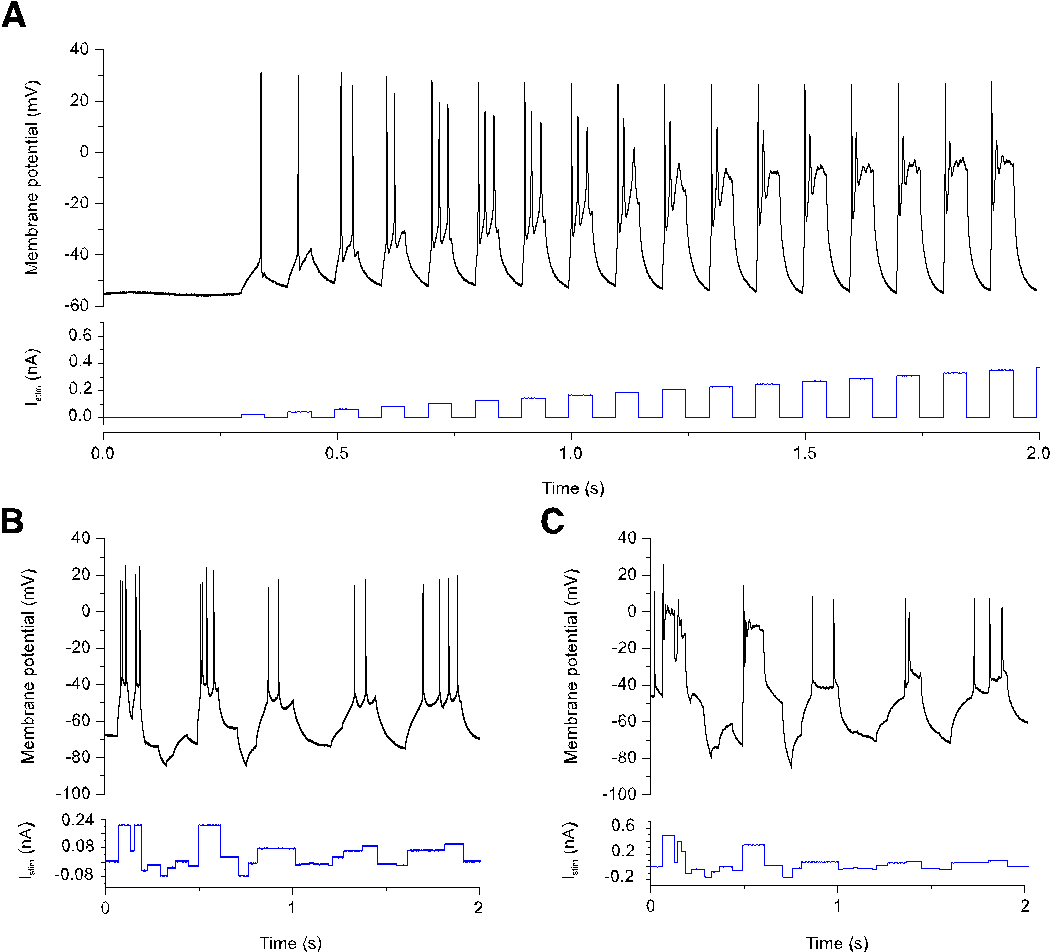


**Figure S2. Action potentials under stimulation by current steps of increasing amplitude**

(**A**) A calibrating current protocol consisting of consecutive step currents of increasing amplitude was used to determine the current range suitable for data assimilation. The amplitudes of current steps in the second half of this particular calibration induce depolarization block, which reduces the amount of information that can be retrieved regarding the underlying ion-channel dynamics. (**B**) A suitably calibrated stimulation protocol using intermediate stimulation currents. (**C**) Exemplar portion of a protocol using large amplitude stimulation currents.

**
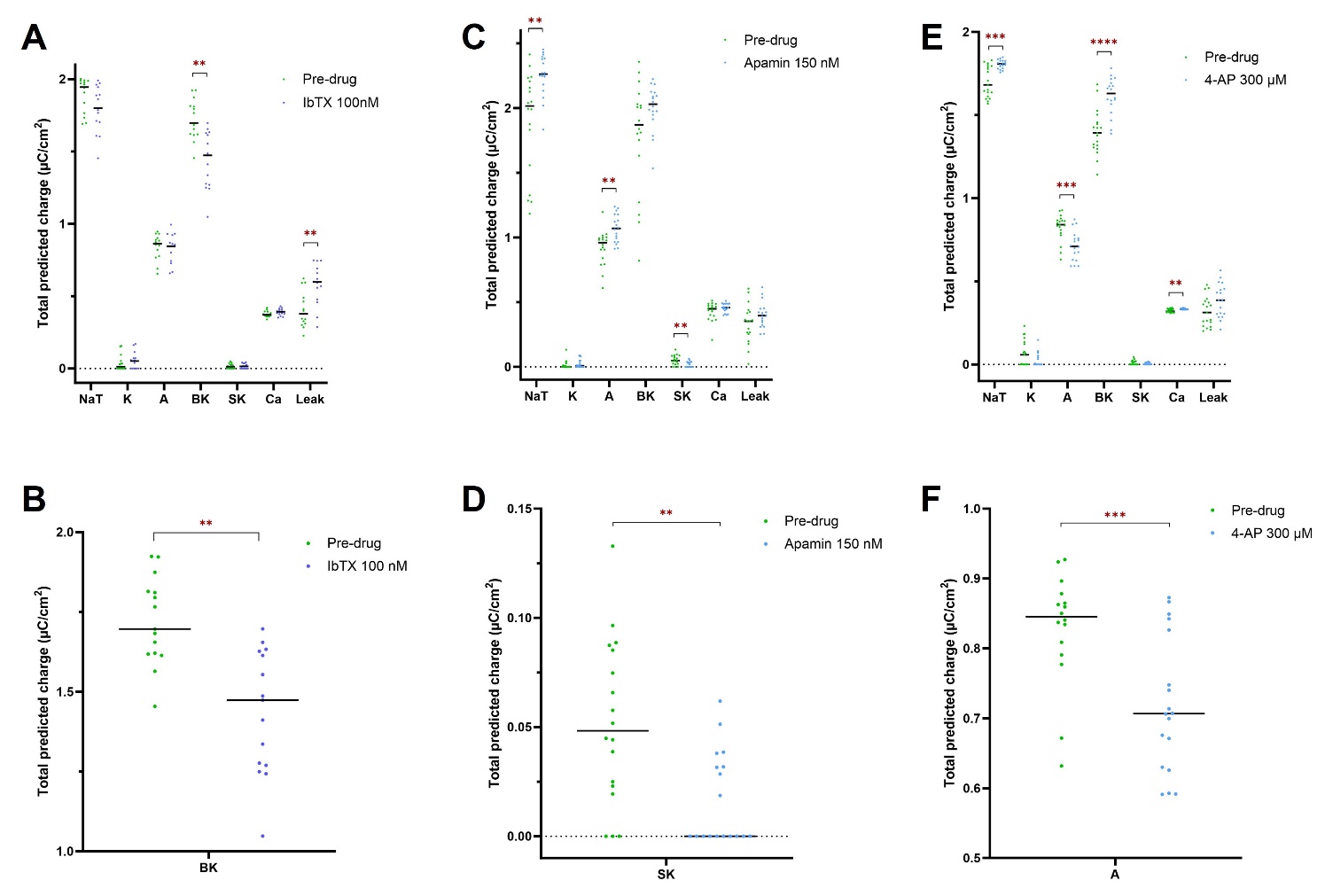
**

**Figure S3. Total estimated charge transferred per ion channel before and after channel inhibition**.

(**A**) Total predicted charge integrated across the 2000 ms window of Figs. S1A, S1B for each channel. The green dots (*N*=15) show the charge transferred pre-drug for the NaT, K, SK, BK, A, Ca, Leak channels. The blue dots (*N*=15) show the change in charge transferred after the application of 100 nM Iberiotoxin (BK blocker). (**B**) BK detail only. (**C**) Total ion charge transferred across the 2000 ms window inferred from pre-drug data (green dots, *N*=18) and after applying 150 nM apamin (SK blocker; blue dots, *N*=18) for all channels. (**D**) SK ion channel detail only. (**E**) Total ion charge transferred across the 2000 ms window inferred from pre-drug data (*N*=19), and after 300 µM 4-AP (*N*=18) for all channels. (**F**) A-type channel detail only. Horizontal lines represent median values. The asterisk pair represents multiplicity adjusted q values from multiple Mann-Whitney U tests using a False Discovery Rate approach (Q) of 1%.

**
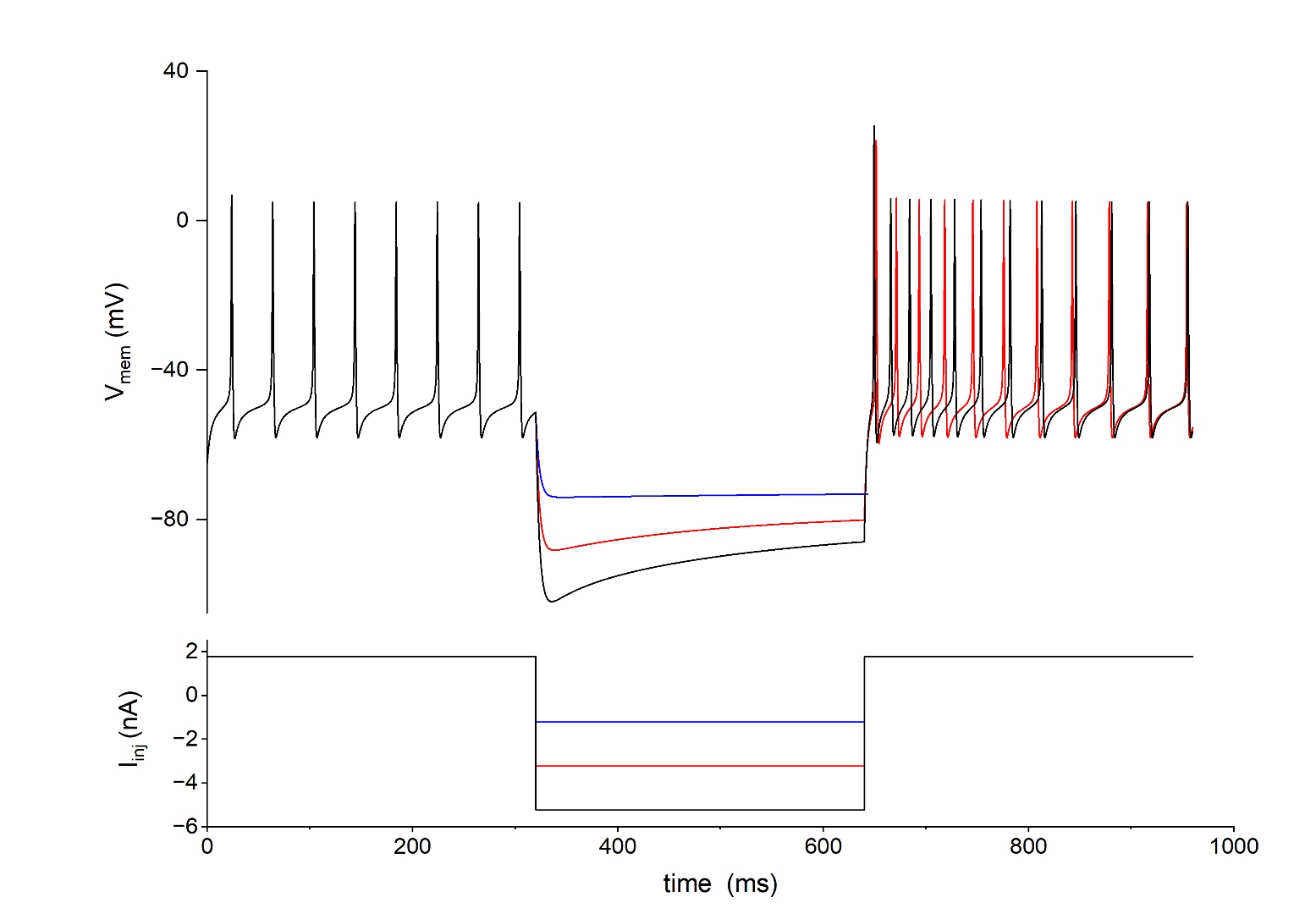
**

**Figure S4. HCN “sag” current predicted by the completed model**.

The ‘sag’ current increases with increasingly negative hyperpolarizing current step. The model completed with the estimated parameters is verified to be consistent with the expected dynamics of the HCN channel.
